# Motor patterns of patients with spinal muscular atrophy suggestive of sensory and corticospinal contributions to the development of locomotor muscle synergies

**DOI:** 10.1152/jn.00513.2022

**Published:** 2024-01-17

**Authors:** Vincent C. K. Cheung, Sophia C. W. Ha, Janet H. Zhang-Lea, Zoe Y. S. Chan, Yanling Teng, Geshi Yeung, Lingqian Wu, Desheng Liang, Roy T. H. Cheung

**Affiliations:** ^1^School of Biomedical Sciences, and Gerald Choa Neuroscience Institute, The Chinese University of Hong Kong, Hong Kong, China; ^2^Department of Health and Physical Education, The Education University of Hong Kong, Hong Kong, China; ^3^School of Nursing and Human Physiology, Gonzaga University, Spokane, Washington, United States; ^4^Faculty of Kinesiology, University of Calgary, Calgary, Alberta, Canada; ^5^State Key Laboratory of Medical Genetics and School of Life Sciences, Central South University, Changsha, Hunan, China; ^6^Department of Psychology, University of Pennsylvania, Philadelphia, Pennsylvania, United States; ^7^School of Health Sciences, Western Sydney University, Sydney, New South Wales, Australia; ^8^Joint Laboratory of Bioresources and Molecular Research of Common Diseases, The Chinese University of Hong Kong and Kunming Institute of Zoology of the Chinese Academy of Sciences, Hong Kong, China

**Keywords:** locomotion, motor development, muscle synergy, nonnegative matrix factorization, spinal muscular atrophy

## Abstract

Complex locomotor patterns are generated by combination of muscle synergies. How genetic processes, early sensorimotor experiences, and the developmental dynamics of neuronal circuits contribute to the expression of muscle synergies remains elusive. We shed light on the factors that influence development of muscle synergies by studying subjects with spinal muscular atrophy (SMA, types II/IIIa), a disorder associated with degeneration and deafferentation of motoneurons and possibly motor cortical and cerebellar abnormalities, from which the afflicted would have atypical sensorimotor histories around typical walking onset. Muscle synergies of children with SMA were identified from electromyographic signals recorded during active-assisted leg motions or walking, and compared with those of age-matched controls. We found that the earlier the SMA onset age, the more different the SMA synergies were from the normative. These alterations could not just be explained by the different degrees of uneven motoneuronal losses across muscles. The SMA-specific synergies had activations in muscles from multiple limb compartments, a finding reminiscent of the neonatal synergies of typically developing infants. Overall, while the synergies shared between SMA and control subjects may reflect components of a core modular infrastructure determined early in life, the SMA-specific synergies may be developmentally immature synergies that arise from inadequate activity-dependent interneuronal sculpting due to abnormal sensorimotor experience and other factors. Other mechanisms including SMA-induced intraspinal changes and altered cortical-spinal interactions may also contribute to synergy changes. Our interpretation highlights the roles of the sensory and descending systems to the typical and abnormal development of locomotor modules.

**NEW & NOTEWORTHY** This is likely the first report of locomotor muscle synergies of children with spinal muscular atrophy (SMA), a subject group with atypical developmental sensorimotor experience. We found that the earlier the SMA onset age, the more the subjects’ synergies deviated from those of age-matched controls. This result suggests contributions of the sensory/corticospinal activities to the typical expression of locomotor modules, and how their disruptions during a critical period of development may lead to abnormal motor modules.

## Graphical abstract

**Figure:**
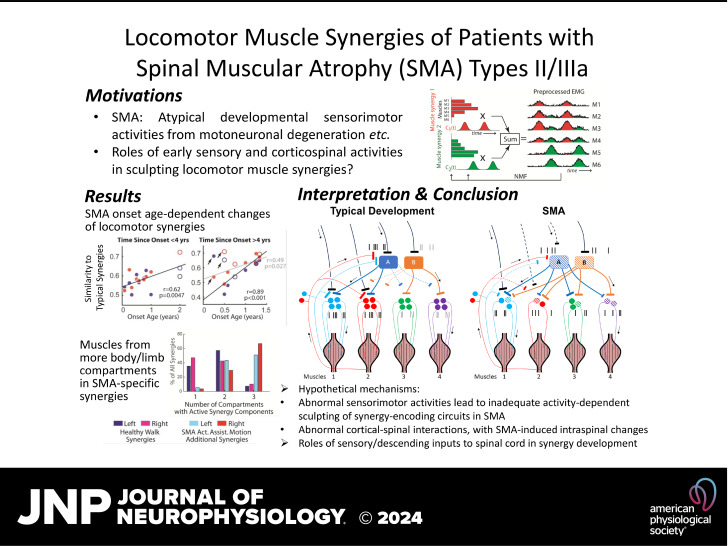

## INTRODUCTION

**Figure 1.**
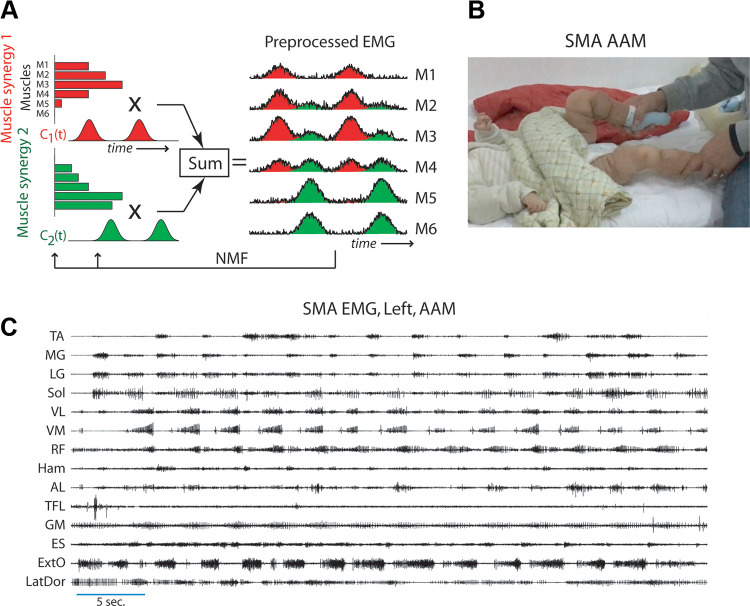
Concepts invoked in this study. *A*: a schematic illustrating the definition of muscle synergy. Each synergy (red or green bars) is a time-invariant activation balance profile across the set of recorded muscles (M1 to M6), and is activated, through multiplication, by a time-varying coefficient (*C*_1_(*t*) and *C*_2_(*t*)). The variability of the preprocessed electromyographic data (EMGs) is then explained by the linear summation of the waveforms contributed by the different synergies. Both the synergies and their coefficients can be extracted from EMG using the nonnegative matrix factorization algorithm (NMF). *B*: a snapshot showing the performance of active-assisted motion (AAM) in a subject with spinal muscular atrophy (SMA). Many of our subjects with SMA did not possess the ability to walk independently; for them we could at best record EMGs during cycling-like AAM, performed with the subject lying supine and with assistance from a physical therapist. *C*: an example of raw surface EMGs of 14 leg and trunk muscles recorded from a subject with SMA during AAM. TA, tibialis anterior; MG, medial gastrocnemius; LG, lateral gastrocnemius; Sol, soleus; VL, vastus lateralis; VM, vastus medialis; RF, rectus femoris; Ham, hamstrings; AL, adductor longus; TFL, tensor fascia latae; GM, gluteus maximus; ES, erector spinae at L2; ExtO, external oblique; LatDor, latissimus dorsi.

The complexity of the neural control of locomotion has continued to baffle motor neuroscientists (1). The diverse motor patterns associated with robust locomotor control are likely generated by the flexible combination of discrete neuromotor modules representable as muscle synergies, each of which activates a group of muscles according to a certain temporal profile (Fig. 1*A*), thereby ensuring the spatiotemporal coordination of muscle activities (2–6). Locomotor muscle synergies identified from multi-muscle electromyographic data (EMGs) using factorization algorithms (7) have been linked to either the activities (8–10) or connectivity (11) of spinal premotor interneurons, and hence may be representations of neurophysiological entities utilized by the central nervous system (CNS) for locomotor control (12).

Although the neural origin of the locomotor synergies has been argued for and demonstrated (13–15), there has also been growing interest in understanding their ontological origin (16). It remains obscure to date which aspects of the synergies and their activations originate from genetic processes, early neuronal or sensorimotor activities exploited by neurodevelopment for neuronal network pruning or optimization, other experiences later in life, or any combinations of the above. One possibility is that the modular structures used by adults emerge soon after birth and are determined early in development through innate genetic programs (17) and/or spontaneous in utero motor activities (18, 19). Indeed, human neonates of a few days old exhibit kicklike air stepping that is underpinned by synergy temporal activation patterns reminiscent of those in adult walking (20). The same temporal patterns appear in the locomotor behaviors of multiple bird and mammalian species (21), suggesting that they may be encoded by ancestral motor networks specified by evolutionarily conserved genes (22). Recent data obtained from spinalized rats likewise indicate that neonatal synergies for walking are preserved into adulthood (23).

Another view of locomotor development accords postnatal and infantile sensorimotor experience a more prominent role in the shaping of locomotor patterns. The developing CNS may arrive at the adult muscle synergies through a period of motor exploration during which many seemingly random muscle patterns are tried, and the adaptively valuable ones are detected through sensory afferent signals and then retained (24, 25). Consistent with this theory, motor outputs of human infants before walking onset are highly variable (26, 27), thereby providing the raw materials for this proposed pattern selection (28, 29). With this developmental mechanism, the synergies thus discovered can automatically match with the biomechanical properties of the growing limbs for effective control and better realization of task goals (30–32). It also echoes the recent idea that during development and motor learning, the CNS acquires novel muscle synergies by relying on exploratory searches of reward-generating motor patterns (33, 34).

A third view of locomotor development emphasizes the contributions from corticospinal activities early in life. In humans at birth, the spinal gray matter is already innervated by the corticospinal system, but through the first 2 yr of life, the descending axons continue to undergo myelination and refinement of their projection patterns through activity-dependent mechanisms (35). Concurrently, activities of the corticospinal system and the sensory afferents together direct spinal plasticity to shape and configure the spinal sensorimotor circuitries (36–38), including the reflex pathways (39) and likely, the circuits that organize the locomotor synergies. In human toddlers, the temporal activations of the muscle synergies that emerge around walking onset cohere with motor cortical activities (40), a finding suggestive of cortical involvement in the maturation and activation of those synergies. The corticospinal pathways may even cooperate with the spinal circuits in structuring the locomotor modules. In the adult rat, the locomotor synergies after their complete spinal transection are more similar to the synergies of other adults that are transected as neonates than their own synergies before transection (23), raising the possibility that during development, supraspinal regions may fine-tune the structures of the muscle synergies encoded within the spinal network (23, 41).

The views of locomotor development outlined earlier are certainly not mutually exclusive. It is possible that neonates are born with a rudimentary version of the locomotor synergies that are then sculpted by sensory afferent and descending corticospinal activities from the exploratory and voluntary motor activities to become the mature synergies of the adults. In human neonates, for instance, stepping elicited on treadmill is generated by two nonsparse muscle synergies (21) that coactivate many leg muscles (26, 42, 43). During early locomotor development, these immature synergies fractionate into sparser synergies with fewer muscles; these fractionations are then activated by the same temporal patterns already deployed in neonatal kicks to become generators of independent walking in toddlers and preschoolers (20). It is not unreasonable to suppose that fractionation of the inborn synergies during walking development is driven by the individual’s sensorimotor experience and activities of the descending system between birth and the walking onset and beyond. Indeed, patterns of synergy fractionation during the development of running displayed some intersubject variability (see Supplemental Table S2 of Ref. 32), possibly reflecting the varied motor experience of the subjects.

In this study, we aim to examine the roles of early sensorimotor experience and descending neural activities in the expression of muscle synergies during human locomotor development. Although previous studies have hinted at sensory and corticospinal contributions to muscle synergy development through recordings from typically developing infants and children (21, 27, 32, 40, 44), these contributions have never been explicitly demonstrated with manipulations of the sensory and/or descending inputs to the spinal cord during development. Such manipulations can be achieved in animal models by interfering with the animals’ developmental experience (45) or neural pathways (38), but for humans we can only rely on subjects with atypical motor histories during the presumed critical period (46) or the presence of a movement disorder.

Here, to achieve our goal, we study children with spinal muscular atrophy (SMA) types II or IIIa, an autosomal recessive genetic disorder characterized by progressive degeneration of primarily α-motoneurons due to mutations in the survival motor neuron 1 gene (*SMN1*), which encodes the survival motor neuron (SMN), a protein critical for the motoneurons’ survival (47). In patients with these milder SMA types, onset of motor symptoms occurred between 2 mo and 3 yr of age. They can maximally achieve independent sitting, though some manage to stand alone or minimally walk with assistance or independently (48, 49); thus, their sensorimotor histories between birth and the typical age of walking maturation differ dramatically from those of the typically developing children. Also, although SMA is classically described as a lower motor neuron disorder associated with the degeneration of motoneurons (50–52) and elimination of the synapses on them from the primary sensory afferents (53), it has been increasingly recognized that the motor cortex and cerebellum may also be affected even in milder SMA forms (54). Case reports have revealed reduced numbers of motor cortical neurons and myelinated corticospinal axons in type II patients (55) and degeneration of cerebellar Purkinje and granule cells in type III patients (56). In a mouse model of severe SMA, by the middle symptomatic stage, electrophysiological abnormalities were observed in both layer V pyramidal and Purkinje cells, with the reduced excitability of both cell types by the late disease stage being the most salient dysfunction (57). The exact origin, time courses, and functional implications of these nonmotoneuronal abnormalities have remained obscure, especially for the less severe SMA types considered here. But given the aforementioned findings, in patients with SMA types II/IIIa, after disease onset the corticospinal inputs to the spinal cord are probably altered in some ways, either directly through changes within the motor cortical network, or indirectly through changes of cerebellar modulations of motor cortical activities.

Within the spinal cord, SMN depletion leads specifically to losses of α-motoneurons with the γ-motoneurons and interneurons spared from degeneration, a loss pattern found even in a mouse model of severe SMA (58). Consistent with the observed sparing of spinal interneurons, the isolated spinal cord of mice with severe SMA can be stimulated to produce locomotor-like activities at the earliest symptomatic stage (59). Taken together, these studies suggest that deviations of the SMA synergies from the normative should exhibit a change pattern attributable either to abnormal synergy development from the subjects’ atypical sensorimotor experiences and/or abnormal descending inputs to the spinal cord, to any possible changes of the properties of the synergy-encoding spinal interneurons induced centrally by motoneuronal losses and/or intraneuronal SMN depletion (60), to any compensatory motor patterns used by the subjects, or to the combined consequences of these factors.

We therefore seek to characterize the locomotor synergies of SMA children and compare them with those of age-matched typically developing subjects to derive insights into the abnormal and normal development of muscle synergies. Specifically, we hypothesize that the SMA subjects’ locomotor synergies are different from the normative in that they are abnormal or incomplete fractionations of the precursor locomotor synergies, and that the pattern of differences across subjects would suggest contributions from sensory afferent and descending inputs to the spinal cord in the synergies’ developmental expression. We recruited subjects with SMA with disease onset age of 0.5 to 2 yr, a range that overlaps with the period of typical developmental synergy fractionation (20, 21). To determine the synergies, we identified a motor task suitable for both ambulatory and nonambulatory SMA subjects that should offer access to the locomotor muscle synergies, and recorded bilateral multi-muscle EMGs from them during this task. We found that the earlier the SMA onset age, the more different the muscle synergies were from the normative, a finding consistent with the idea that early motor experience and/or corticospinal activities play a role in the proper developmental expression of the locomotor synergies.

## MATERIALS AND METHODS

### Subjects and SMA Diagnosis

Subjects with confirmed diagnosis of spinal muscular atrophy (SMA) (*n* = 17; 7 males, 10 females; age of 5.94 ± 4.71 yr old [means ± SD], range of 1.95–20.5) were recruited from The Hunan Jiahui Genetics Hospital, State Key Laboratory of Medical Genetics, Changsha, China (*n* = 16) or the Heep Hong Society of Hong Kong (*n* = 1). The disease onset age (0.08–2 yr old) was retrieved from the subject’s medical record, or if this information was not available in the record, determined by interviewing the subject and/or the subject’s guardians. Diagnosis of SMA was based on assays of the copy numbers of the *survival motor neuron 1* (*SMN1*) and *survival motor neuron 2* (*SMN2*) genes using multiplex ligation-dependent probe amplification (MLPA) (61). Briefly, genomic DNA was extracted from 3 to 4 mL of peripheral venous blood (EDTA anticoagulant) using the standard phenol-chloroform method. The SALSA MLPA P060/P021 SMA kit (MRC Holland, Amsterdam, The Netherlands) was used to determine the copy number of exon 7 and 8 in the *SMN1* and *SMN2* genes. All products were examined by an ABI 3100 genetic analyzer (Applied Biosystems, Carlsbad, CA), and MLPA analysis was performed following the manufacturer’s instructions (62).

Besides, we enrolled two groups of healthy subjects with different age ranges (Children group, *n* = 13, age 3.66 ± 2.68, range of 2.02–11.01; Adult group, *n* = 8, age 20.45 ± 1.28, range of 19–22.7), serving as age-matched controls for two subgroups of SMA subjects at different ages (Children group, *n* = 16, age 5.09 ± 3.08, range of 1.95–12; Adult group, *n* = 1, age 20.5), respectively. For both age groups, the average ages of the healthy subjects and subjects with SMA were not statistically different (Children group, *P* = 0.086, Mann–Whitney; Adult group, *P* = 0.92, one-sample *t* test). In addition, two healthy children (*CH1* and *CH2*, age 9.1 and 13.6) were separately recruited for providing supplementary data that assess the potential differences in the muscle synergies of two behaviors—overground walking and active-assisted motion—that were performed by the healthy and SMA subjects in the experiment (see *Behavioral Tasks*). They were not included in the aforementioned subject groups. All control subjects had no history of neurological, psychiatric, or musculoskeletal disorders.

All procedures were approved by The Joint Chinese University of Hong Kong-New Territories East Cluster Clinical Research Ethics Committee (No. 2017.690), The Departmental Research Committee of the Department of Rehabilitation Sciences, The Hong Kong Polytechnic University (No. HSEARS20150619001), and The Ethics Committee of The Jiahui Genetics Hospital (No. 2019031501). Written informed consent was obtained from all participants or their guardians (for subjects under age 18) before experimentation. The demographic, genetic, and clinical data of the enrolled subjects with SMA are summarized in Table 1.

**Table 1.** The demographic, genetic, and clinical data of the enrolled subjects with SMA

Age Group	Subject Number	Age, yr	Onset Age, yr	Time since Onset, yr	Gender	Height, cm	Weight, kg	Gene Copy Numbers				Revised Hammersmith Scale, Left (Right)	Motor Tasks
								SMN2 Exon 7	SMN2 Exon 8	SMN1 Exon 7	SMN1 Exon 8		
Children	1501(1)+	2.12	2	0.12	M	83	9.53	NA	NA	NA	NA	NA	W
	1501(2)+	2.56	2	0.56	M	NA	NA	NA	NA	NA	NA	NA	W
	1605	11.5	0.5	11	F	118	20.5	3	3	0	0	NA	W
	1701	5.48	0.58	4.9	M	117	26.89	3	3	0	0	6.5 (8.5)	A
	1702	3.67	0.5	3.17	M	95	13.71	3	3	0	0	1.5 (2)	A
	1703	12.0	0.5	11.5	F	128	19.96	3	3	0	0	9	A
	1704	5.92	0.25	5.67	F	107	15.84	3	3	0	0	9	A
	1705	3.77	0.33	3.43	F	95	13.68	3	3	0	0	2	A
	1706	2.01	0.08	1.93	M	79	9.27	3	3	0	0	3	A
	1710	3.67	0.58	3.08	M	87	16.4	3	3	0	0	1	A
	1711	7.50	1.25	6.25	F	130	37.63	3	3	0	0	40.75 (41)	W, A
	1712	7.50	1.33	6.17	F	116	25.06	2	2	0	0	48	W, A
	1713	2.92	0.83	2.08	F	88	11.31	3	3	0	0	8.5	A
	1714	1.95	0.25	1.7	M	83	9.49	3	3	0	0	0	A
	1715*	3.58	1	2.58	F	90	13.2	3	2	0	1	13	A
	1716	3.83	0.75	3.08	M	96	16.65	3	3	0	0	4.75	A
	1717*	6.50	1	5.5	F	108	17.85	3	2	0	1	17.5	A
Adult	1602	20.5	0.75	19.75	F	142	40	3	3	0	0	NA	W**

A, active assisted cycling-like motion; SMA, spinal muscular atrophy; W, walking. +,Two sessions of data were collected from subject 1501. Number in parentheses refers to session number; *in *subjects 1715* and *1717*, it is likely that in the one remaining copy of *SMN1*, a mutation in exon 7 converts it into one that resembles that for *SMN2*. This converted exon 7, when linked to the original exon 8, produces a hybrid *SMN2* (exon 7)-*SMN1* (exon 8) that should in turn produce SMN2-like proteins. Their *SMN2* copy number should therefore effectively be 3; **subject walked with crutch support.

### Motor Functional Assessment of Subjects with SMA

In 14 of the 17 subjects with SMA, the residual physical motor abilities of the subjects with SMA were assessed by the Revised Hammersmith Scale (RHS), a scoring system specifically designed for SMA (63). Included in this score are 36 test items grouped under 15 motor functional categories (including sitting, supine, rolling, prone, standing, run/walk, etc.). For each item, a score of 0, 1, or 2 can be awarded depending on performance, thus making a maximum total RHS of 72. The lower the RHS, the lower the overall motor capability. When performing the RHS tests, we found it necessary to score item no. 2 (hands to head in sitting) for the left and right sides separately; thus, for some subjects, the total RHS for the two sides were slightly different. All RHS scores were assessed by a registered physiotherapist immediately after electromyographic data (EMGs) were recorded (see *EMG Recordings and Preprocessing*).

### Behavioral Tasks

Among the 17 subjects with SMA, only five had sufficient residual motor function and strength to walk either unsupported or with walking aids. For the rest, to elicit locomotor-like lower-limb motor patterns, they were instructed to perform an active-assisted motion (AAM) task, as follows. The subject lied supine on a bed while both legs were lifted by a physiotherapist standing beside the bed, holding the subject’s ankles. The supine subject was then encouraged to produce lower limb cycling-like motion at self-selected cycle frequency while the therapist concurrently provided assistive forces by following the subject’s ankle trajectory with his hands (Fig. 1*B*). The resulting leg motion thus loosely resembled those observed in some lower limb rehabilitation based on assisted supine cycling (64). Assisted motion was performed with each leg alone, and with both legs alternating.

For the high-functioning SMA subjects able to walk and all healthy controls, each subject walked along a 3- to-8 m walkway at self-selected preferred speed for multiple times. For safety, one subject with SMA walked with crutch support. In each subject, data from a minimum of 22 step cycles were included in our muscle synergy analysis (65) (see *Muscle Synergy Extraction and Clustering*). In two subjects with SMA, data from both unaided walking and AAM were recorded. In one subject with SMA, two sessions of walking (∼5 mo apart) were conducted; thus, six sessions of walking from five subjects with SMA were analyzed.

For the additional healthy subjects *CH1* and *CH2*, EMGs were collected during both overground walking at self-selected preferred speed and AAM. They were instructed to perform AAM at a cycle frequency that matched the cadence of their walking episodes.

### EMG Recordings and Preprocessing

During trials of walking or active-assisted motion, surface EMGs were recorded from 28 lower limb and trunk muscles (14 muscles on each side) by two synchronized wireless EMG systems (Trigno, Delsys, Boston, MA) at 2,000 Hz. The recorded muscles included tibialis anterior (TA), medial gastrocnemius (MG), lateral gastrocnemius (LG), soleus (Sol), vastus lateralis (VL), vastus medialis (VM), rectus femoris (RF), hamstrings (Ham), adductor longus (AL), tensor fascia latae (TFL), gluteus maximus (GM), erector spinae at L2 (ES), external oblique (ExtO), and latissimus dorsi (LatDor) (Fig. 1*C*). For *CH1* and *CH2*, only the right-sided muscles were recorded. Before placing the EMG sensors, the relevant skin surfaces were cleaned with alcohol. The sensors were attached according to guidelines of the Surface Electromyography for the Non-Invasive Assessment of Muscles—European Community project (SENIAM), and placed at the muscle bellies in the direction of the muscle fibers for optimal signals. White surgical tape (3M Transpore) and self-adherent bandage wrap (3 M Coban) were then used to mechanically stabilize the sensors to reduce motion artifact in the recorded signals. For every trial, data were acquired using the EMGworks Acquisition Software (Delsys, Boston, MA). Raw EMG data files were then exported as .txt files for further analysis using customized scripts in Matlab (MathWorks, Natick, MA).

The EMG signals were preprocessed by standard and customized Matlab functions as described previously (66). Raw EMGs were high-pass filtered (window-based finite impulse response [FIR] filter, 50th order; cutoff frequency of 50 Hz) to remove motion artifact, baseline-subtracted for DC-offset correction, rectified, low-pass filtered (window-based FIR filter, 50th order; cutoff frequency of 20 Hz) to remove noise, then integrated over 20-ms intervals. Occasional high-amplitude spikes caused by noise observed after filtering were smoothened with spline interpolation. The EMG amplitude of each muscle was then normalized to unit variance.

### Muscle Synergy Extraction and Clustering

For each side of every subject and behavior, muscle synergies were extracted from the preprocessed EMGs using the standard version of the nonnegative matrix factorization algorithm (NMF) (67, 68) (Fig. 1*A*), which models the EMG data matrix **D** to be a linear combination of a set of time-invariant muscle synergies (**W**) scaled by their corresponding time-varying coefficients (**C**). To determine the number of muscle synergies (i.e., dimensionality), we first successively increased the number of synergies extracted from 1 to 14 (the number of muscles recorded on each side). At each number, synergy extraction was repeated 20 times, each time with different randomized matrix initializations that were uniformly distributed between 0 and the maximum observed EMG amplitude, and the repetition yielding the highest EMG reconstruction *R*^2^ (the “best” repetition) was noted. Then, the number of muscle synergies whose best repetition yielded an *R*^2^ that was closest to 80% was selected as the dimensionality of the EMGs. Here, when calculating the *R*^2^, the sum of squares total (SST) was defined as the total squared difference between each data point of each muscle and the data mean of that muscle (32, 69, 70). Note that this definition of *R*^2^ differs from some definitions of the Variance Accounted For that use an SST derived without subtraction of data mean (71).

To clarify the major categories of muscle synergies present across subjects, the synergy vectors from each subject group and motor behavior were grouped into clusters using the *k*-means algorithm, implemented using the Matlab function kmeans (option of squared Euclidean distance; number of replicates = 5,000). The optimal number of clusters was determined by finding the number that yielded the maximum average silhouette value.

### Muscle Synergy Comparison

#### Age considerations.

Our aim is to characterize how locomotor muscle synergies in subjects with SMA differ from those in healthy subjects at similar ages. Given that one high-functioning SMA subject with valuable walking EMGs was considerably older than the rest of the subjects with SMA (Table 1, *subject 1602*), we recruited two groups of controls at different ages so that the age of the older controls (Adult group) could be matched to that of the older subject with SMA while the age of the younger controls (Children group), to those of the rest. In all SMA and healthy subjects EMGs from both sides were collected, thus allowing us to also match the side in our control-versus-SMA comparisons.

#### Behavioral considerations.

Another consideration regards the reality that most of our subjects with SMA could not walk, and hence we could only record from them EMGs during cycling-like active-assisted motion (AAM) (see *Behavioral Tasks*). To evaluate whether the AAM muscle synergies are reasonable proxies of the walking synergies in subjects with SMA, we implemented two analyses. The first analysis assessed the similarity between the normative walking synergies of the healthy subjects and those of AAM and walking of two ambulatory subjects with SMA from whom data during both behaviors were collected (*subjects 1711* and *1712*, Table 1). To evaluate this similarity, the muscle synergies of each age- and side-matched limb in the control group were fit to the AAM and walking EMGs of these SMA limbs, respectively, with the quality of fit indicated by the EMG reconstruction *R*^2^ (see *Synergy comparison by cross-dataset synergy fitting*). To extend this validation to the nonambulatory subjects with SMA, in the second analysis, we assessed the similarity between the normative walking and AAM synergies of healthy subjects and those of AAM in all subjects with SMA. The walking and AAM synergies of the healthy *CH1* and *CH2* were, respectively, fit to the AAM EMGs of all SMA limbs. In both analyses, a positive, significant correlation between the two sets of *R*^2^s derived (from fitting to walking vs. AAM SMA EMGs in the first, and from fitting with walking vs. AAM healthy synergies in the second) across the control-SMA limb pairs would suggest that the AAM EMGs of subjects with SMA contain information on how similar their walking-related synergies are to the normative walking synergies (for more detailed argument, see Supplemental Text; all Supplemental material is available at http://doi.org/10.6084/m9.figshare.24978804).

#### Synergy comparison by cross-dataset synergy fitting.

Throughout this work, to address our questions we needed to compare the muscle synergy sets of either different subjects (e.g., healthy vs. subjects with SMA) or different behaviors (e.g., walking vs. AAM), and to quantify their similarity. One obvious approach of comparison is to match the individual synergies of the two sets by a goodness-of-fit measure (e.g., scalar product). But, as has been pointed out earlier (72), any apparent dissimilarity between synergy sets could arise from different representations of the same EMG subspace discovered by NMF. Thus, a more comprehensive approach of synergy comparison is to quantify the similarity between the EMG subspaces (within the muscle space) rather than that between individual basis vectors of the subspaces. Following earlier works (32, 34, 69), we achieved EMG subspace comparison by evaluating the extent to which the synergies extracted from the EMG of one subject/behavior could generalize to describing the EMG of another subject/behavior. Specifically, the muscle synergies extracted from a subject/behavior (EMG set “A”) were fit to the EMG of another subject/behavior (EMG set “B”) with goodness-of-fit quantified by the EMG reconstruction *R*^2^. If the two data are indeed generated by the same underlying muscle synergy set, the *R*^2^ of this fit (describing “B”) should be similar to the *R*^2^ of the original extraction (describing “A”). Fitting a set of muscle synergies (**W_fit_**) to another set of EMG was achieved by the NMF: precisely, by implementing just the NMF update rule for **C** with **W** fixed at **W_fit_** across all iterations while skipping the update rule for **W**.

More specifically, when comparing the control and SMA muscle synergies at subject group level, since within each group there is interindividual variability in the synergies, any muscle synergy difference between the two groups is significant only if such difference is beyond that expected from the variability within the controls. In our comparison, we first fit the synergies of each control subject to the EMG of every other control subject to generate a set of baseline EMG-reconstruction *R*^2^ values that reflect the within-control synergy variability. We then fit the synergies of each control to the EMG of every subject with SMA to result in a second set of *R*^2^ values. An *R*^2^ set from the control-to-SMA fits that is statistically lower than the control-to-control *R*^2^ set would mean that the healthy synergies, on average, cannot generalize to describing the SMA EMGs as well as the EMGs from other healthy subjects, thus implying that the muscle synergies of the control and SMA groups are significantly different (69). For each individual SMA subject, the difference between the subject’s synergy set and those of the control subjects as a group is then conveniently measured by the average *R*^2^ values across the fits with the synergies of all controls.

#### Synergy fitting with concurrent synergy updating.

Although the aforementioned comparison of *R*^2^ values from synergy fitting indicates whether the muscle synergies of the control and SMA groups differ, it conveys no information on which muscle synergies in the control group may be changed in the SMA group, nor whether any between-group synergy difference may be due to the presence of additional SMA-specific synergies. To obtain this information, we manipulated the NMF so that as **W_fit_** was fit to the EMG, we concurrently updated a prespecified subset of synergies in **W_fit_** (denoted by **W_fit_^up^**, “up” standing for updated, comprising *N^up^* synergies), with the remaining synergies in **W_fit_** enforced to be fixed (**W_fit_^fixed^**), while also extracting extra synergies from the EMG in addition to the original ones being fixed or updated (**W^add^**, “add” standing for additional, comprising *N^add^* synergies). In our control versus SMA comparisons, suppose we have the means (to be described later) to identify *1*) the optimal number of synergies that must be updated (N^up^*), *2*) which of the N^up^* synergies in **W_fit_** should be updated, and *3*) the optimal number of **W^add^** that must be extracted from the SMA data (N^add^*), the control-SMA synergy differences are then indicated by how the N^up^* control synergies in **W_fit_^up^** are altered in the SMA EMG during fitting, and by the N^add^* extracted synergies in **W^add^** which are SMA specific.

The extraction of **W_fit_^up^** and **W^add^** is easily achieved thanks to the component-wise definition of the NMF update rules (67). Without loss of generality, the entire muscle-synergy matrix used in the fitting (**W**) can be written as

$$W\;=\;[{W_{\mathrm{fit}}}^{\mathrm{fixed}}\left|\;{W_{\mathrm{fit}}}^{\mathrm{up}}\right|\;W^{\mathrm{add}}],$$

where [**W_fit_^fixed^** | **W_fit_^up^**] constitutes the original set of synergies from the healthy subject. Updating of **W** can then be restricted to the matrix components within **W_fit_^up^** and **W^add^** while keeping components in **W_fit_^fixed^** constant. Components in **W_fit_^up^** were initialized with their corresponding original synergies, and those in **W^add^**, with random numbers uniformly distributed between 0 and the maximum value in the EMG to be decomposed.

To find which of the original synergies (**W_fit_**) should be included in **W_fit_^up^**, we performed the following steps. Suppose that the original dimensionality of the synergy set is *N^orig^.* For a particular *N^up^*, we repeated the fitting by updating every possible selection of *N^up^* synergies within the *N^orig^* synergies (with a total of *N^orig^*-choose-*N^up^* selections); the combination of *N^up^* synergies yielding the highest *R*^2^ was then selected to be the updated synergy subset.

To find the optimal *N^up^* and *N^add^* (N^up^* and N^add^*) for each subject with SMA, as before we fit the extracted synergies of each control subject first to the EMGs of every other control, and then to the EMGs of every subject with SMA, but this time with concurrent extraction of **W_fit_^up^** and **W^add^**; we then systematically searched for the smallest *N^up^* and *N^add^* in the control-to-SMA fits that resulted in similar *R*^2^s in both the control-to-SMA and control-to-control fits (*P* ≥ 0.05; 2-tailed *t* test for normally distributed samples, or 2-tailed Mann–Whitney test otherwise). More specifically, we began by setting *N^add^* = 0 while successively increasing *N^up^* from 0 to 5 (5 was chosen as the maximum *N^up^* because the dimensionality of the control subjects was ∼6); if none of the *N^up^* fulfilled the statistical criterion for *R*^2^ similarity, *N^add^* was increased to 1 while *N^up^* was again successively increased from 0 to 5. This cycle was repeated, each time with an increase of *N^add^* by 1, until we reached the *N^up^* and *N^add^* that satisfied our *R*^2^ similarity criterion. By increasing *N^up^* before increasing *N^add^* in our search, we guarantee that the EMG of the subject with SMA is still described by the smallest possible dimensionality with the smallest possible number of updated synergies. In all *R*^2^ comparisons, the *R*^2^s from the control-to-control and control-to-SMA fits were compared at identical *N^up^*. Since the dimensionality of the subjects with SMA was on average higher than that of the controls (see results), in all control-to-control fits *N^add^* was kept at 0 while in the control-to-SMA fits *N^add^* was varied, so that the extracted **W^add^** would better reflect the synergies specific to the SMA EMG.

As an example, suppose that the synergies of an SMA subject differ from those in the control subjects (*n* = 13) in that three of the synergies observed in most controls are altered, and there is one synergy unique to this subject with SMA. Then, fitting the 13 control **W_fit_** to this SMA data with *N^up^* = 3 and *N^add^* = 1 should yield *R*^2^s that are similar to or greater than the baseline *R*^2^s from fits among the controls (totaling 13^2^ − 13 = 156 fits), also implemented with *N^up^* = 3.

After implementing the aforementioned fitting procedure on every subject with SMA, the **W_fit_^up^**’s and **W^add^**’s from all fits (with the synergies of the different control subjects) at the SMA subjects’ respective N^up^* and N^add^* were collected together. The pooled **W_fit_^up^** and **W^add^** matrices were then *k*-means clustered separately for downstream characterizations.

### Estimating Motoneuronal Loss in Subjects with SMA

In SMA, the dynamics and extent of motoneuronal functional loss and degeneration are known to be highly uneven across muscles. The pattern of loss across muscles is also highly variable across subjects, with some patients presenting losses first in proximal then in distal limb muscles (49) while others, predominantly in the distal muscles (73). To assess whether the degree of uneven losses across muscles may account for the muscle synergy alterations in subjects with SMA, we estimated the residual motoneuronal number and functions of each muscle from its observed peak EMG amplitude relative to that in healthy controls. The absolute EMG amplitude surely depends on a host of factors, including the number of surviving motoneurons, the shape of the motor unit action potential, and the discharge patterns of motoneurons (74). But given that subjects with SMA tend to have larger single motor unit potentials due to increases in motor unit sizes from compensatory collateral reinnervation (75), any reduction in EMG peak amplitude may reasonably be attributed to losses of motoneurons and/or their impaired discharges, assuming that other experimental variables (e.g., electrode placement positions) stay relatively constant.

For all healthy subjects and subjects with SMA, the peak EMG amplitude of each muscle was obtained by finding the amplitude at 99th percentile of the muscle’s entire record of preprocessed, non-normalized EMG. For each muscle of an SMA limb, its residual function from the remaining motoneurons was then estimated by dividing its peak amplitude by the subject-averaged peak of the same muscle from the side- and age-matched control limbs. A ceiling percentage of 100% was imposed. The reasonableness of using this percentage as a rough proxy of residual motoneuronal number and/or functions was assessed by correlating it with the RHS functional scale of the subjects with SMA. To quantify the degree of uneven motoneuronal losses across muscles, the variance of this percentage across muscles was calculated for each SMA limb. This variance was then further regressed against the similarity between the SMA and control muscle synergies across the subjects with SMA.

### Statistics

To determine whether any two samples may have a difference in mean or median that was statistically significant, either the two-sample *t* test (for normally distributed samples) or Mann–Whitney *U* test (non-normally distributed) was used. Sample normality was assessed using the Lilliefors test. For comparisons of multiple sample groups, the one-way analysis of variance (ANOVA) (normal samples) or Kruskal–Wallis test (non-normal samples) was implemented. For comparisons with *P* < 0.05, this was followed by a post hoc Tukey–Kramer multiple comparison test to identify significantly different group pairs. When assessing the correlation strength between two variables, the Pearson’s correlation coefficient (*r*) was calculated. All statistical tests were implemented in Matlab (Statistics Toolbox in R2019b). Hypotheses were rejected at 5% significance.

## RESULTS

### Genetic and Motor Functional Characterizations of the Subjects with SMA

We recruited 17 subjects with type II or type IIIa spinal muscular atrophy (SMA) and obtained the *SMN1* and *SMN2* copy numbers in 16 of them. None carried any functional *SMN1* copy, but 13 had three copies of *SMN2*, and one had two copies of *SMN2*. In two subjects, for *SMN1* we detected 0 copy of exon 7 and one copy of exon 8, but for *SMN2*, three copies of exon 7 and two copies of exon 8 (Table 1, *subjects 1715* and *1717*). For these two subjects, it is likely that a mutation in exon 7 of an *SMN1* copy converted the exon into one that resembles the exon 7 of *SMN2* (76, 77); this converted exon 7, when linked to the original exon 8, produces a hybrid *SMN2*(exon 7)-*SMN1*(exon 8) that should in turn produce SMN2-like proteins. Their *SMN2* copy number should therefore effectively be 3.

**Figure 2.**
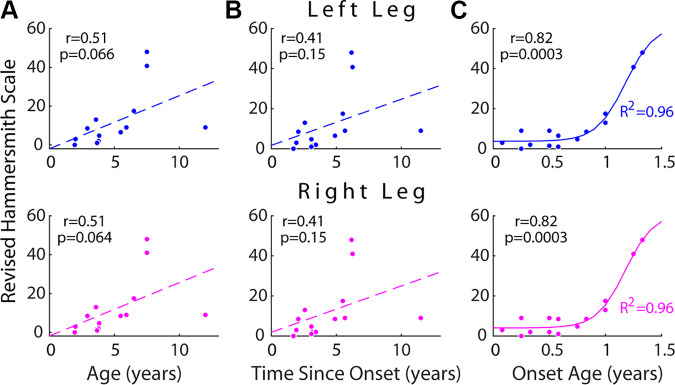
Demographics and motor functional statuses of the enrolled subjects with spinal muscular atrophy (SMA). The residual motor abilities of the subjects with SMA (*n* = 14) were evaluated by the Revised Hammersmith Scale (RHS) (63), with 1 test item scored separately for the left and right sides. The left- and right-side RHS were regressed against the subjects’ age (*A*), the time elapsed since disease onset (*B*), and the age of SMA onset (*C*). The RHS correlated significantly with the onset age (*P* < 0.001), but not with age nor time since onset (*P* > 0.05). For both sides, the RHS-onset age relationship could also be well described by a sigmoidal function: left, RHS = [58.0/(1 + *exp*^−7.7(Onset Age – 1.2)^)] + 3.7 (*R*^2^ = 0.963); right, RHS = [57.2/(1 + *exp*^−7.9(Onset Age – 1.2)^)] + 4.0 (*R*^2^ = 0.959). The Pearson’s *r* values and their associated *P* values (*t* test) of all regressions are shown in the figure. Sigmoidal fitting was performed using Matlab (Curve Fitting Toolbox, Trust-Region algorithm, robust option off).

In 14 subjects with SMA, we evaluated their residual motor functions using the Revised Hammersmith Scale (RHS) (63). In three subjects, the left and right sides were separately scored. Across subjects, the earlier the onset age, the more severe was the motor impairment. For both sides, the RHS correlated significantly with the onset age (*r* = 0.82, *P* = 0.0003; Pearson’s *r* and *t* test; Fig. 2*C*) but not with the subject’s age (left, *P* = 0.066; right, *P* = 0.064; Fig. 2*A*) nor with the time elapsed from disease onset to the time of assessment (*P* = 0.15; Fig. 2*B*). The relationship between RHS and onset age could be well described by a sigmoidal curve (*R*^2^ = 0.96) with the RHS increasing steeply after an onset age of ∼0.8 yr (Fig. 2*C*).

### Muscle Synergies of the Age-Matched Control Subjects

**Figure 3.**
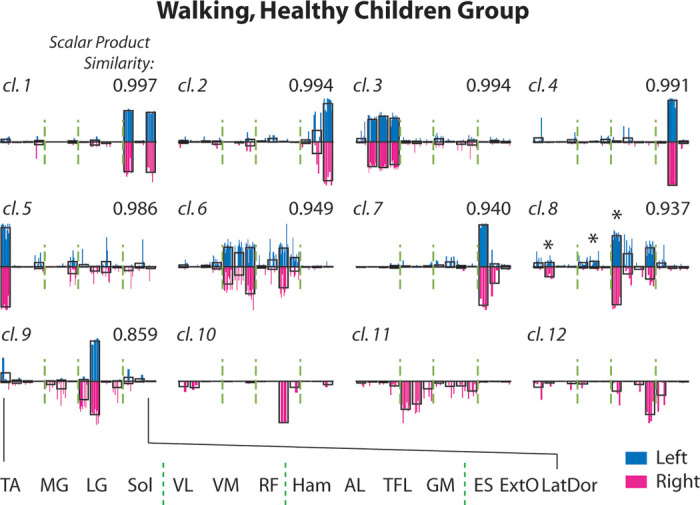
Muscle synergies of the age-matched healthy subjects. As baseline of comparison for assessing the spinal muscular atrophy (SMA) muscle synergies, normative synergies were extracted from electromyographic data (EMGs) recorded from healthy subjects (Children group, *n* = 13) during overground walking. Muscle synergies for the left (*n* = 82) and right (*n* = 89) sides were *k*-means clustered separately, and the left-side clusters were matched to the right-side clusters by scalar product between cluster centroids (value shown at each panel’s *top right*). The individual muscle synergies in each cluster (*cl. 1* to *12*) are shown as colored bars (left, blue; right, magenta) overlaid onto the bars that represent the centroids (uncolored). The left- and right-side normative synergies were highly similar to each other. In all matched clusters, the left and right muscle components were not statistically different (*P* > 0.01, *t* test or Mann–Whitney) except medial gastrocnemius (MG) (*P* = 0.0046, *t* test), vastus medialis (VM) (*P* = 0.0080), and hamstrings (Ham) (*P* = 0.0085) in cl. 8 (*). TA, tibialis anterior; LG, lateral gastrocnemius; Sol, soleus; VL, vastus lateralis; RF, rectus femoris; AL, adductor longus; TFL, tensor fascia latae; GM, gluteus maximus; ES, erector spinae at L2; ExtO, external oblique; LatDor, latissimus dorsi.

We began with a characterization of the muscle synergies of the healthy Children and Adult groups. Since one of the subjects with SMA with valuable walking data was at age 20.5 (Table 1, *subject 1602*), the assessment of this subject demanded a separate Adult control group; all other subjects with SMA were compared against the Children group. These synergies were extracted from EMGs (Fig. 1*A*) collected during overground walking at self-selected preferred speed, and would serve as the baseline of comparison for our age-matched assessment of the synergies from subjects with SMA. In the Children group (*n* = 13), the number of muscle synergies identified from each limb was 6.31 ± 0.63 for the left side, and 6.85 ± 0.80 for the right side. The muscle synergies from all subjects could be grouped by *k*-means into nine clusters for the left side, and 12 clusters for the right (Fig. 3). All left-side clusters could be matched to their corresponding right-side clusters with very high similarity (scalar product between cluster centroids = 0.957 ± 0.047). The three right-side-specific clusters (Fig. 3, *cl. 10*–*12*) were more subject-specific, including synergies from 2, 7, and 4 subjects, respectively. Analogous synergy clusters were observed in the Adult group (*n* = 8), from which we identified nine clusters common to both sides, two for the left only and two for right only. All but one Children-group cluster (*cl. 11* in Fig. 3) could be well matched to an Adult-group cluster.

Our main analytic goal here is to evaluate to what extent the post-SMA locomotor muscle patterns are generated by the normative muscle synergies for walking. To address this question, one experimental difficulty regards the reality that 12 of our 17 subjects with SMA did not possess the ability to walk independently. For these subjects, we could at best record EMGs during cycling-like active-assisted motion (AAM), performed with the subject lying supine and with assistance from a physical therapist (Fig. 1, *B* and *C*). It would therefore be necessary to first evaluate whether we may derive sufficient information about the walking-related locomotor synergies from the AAM muscle patterns of the subjects with SMA, especially since AAM execution may additionally elicit motor patterns from spinal and long-loop reflexes and other joint-protective muscle activities. By fitting the walking synergies of the healthy subjects to the AAM and walking EMGs of ambulatory subjects with SMA (Supplemental Fig. S1*A*; see http://doi.org/10.6084/m9.figshare.24978804 for supplementary text and figure) and then fitting both the walking and AAM synergies of two healthy children to the AAM EMGs of all SMA subjects (Supplemental Fig. S1*B*), we showed that the AAM synergies of both ambulatory and nonambulatory SMA subjects should be synergies related to their potential walking ability (see Supplemental Text). For subjects with SMA, it is therefore justifiable to use their AAM motor outputs for evaluating their walking-related locomotor muscle synergies. Using our NMF implementation, we further confirmed that in healthy subjects, the AAM and walking synergies overlapped substantially though some small differences in synergies between the two tasks related to muscles AL and TFL could be observed (Supplemental Fig. S1, *D* and *E*). In our subsequent comparison of healthy walking and SMA AAM synergies, any synergy differences beyond the ones noted in Supplemental Fig. S1 (*D* and *E*) should originate from synergies specific to SMA rather than the natural AAM-walking behavioral synergy differences observable in all subjects.

### Healthy Synergies Could Not Well Describe EMGs from Early-Onset SMA Subjects

**Figure 4.**
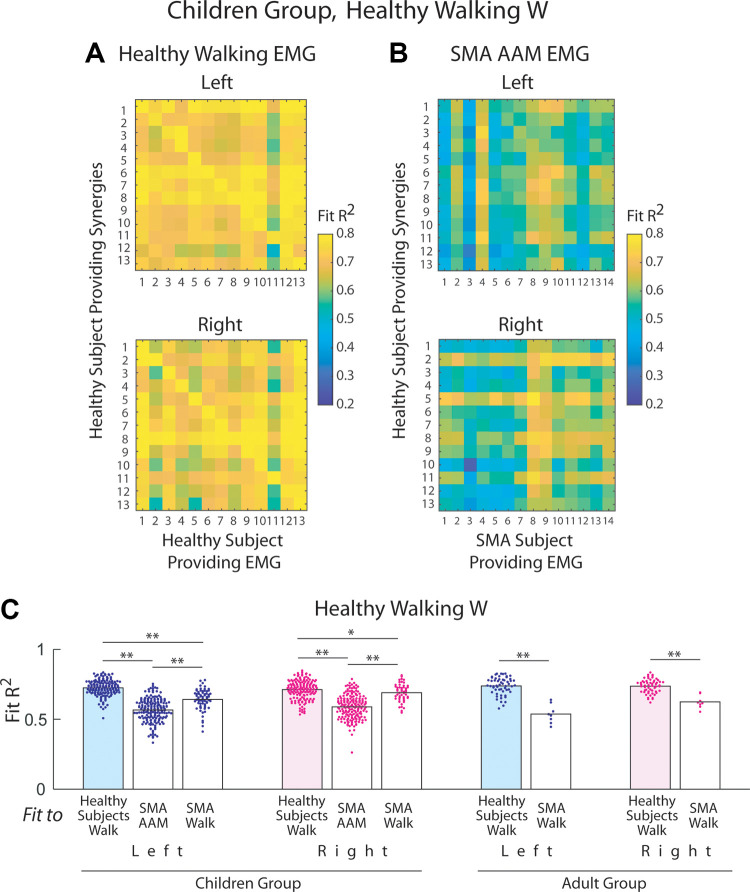
Control muscle synergies could not well-describe electromyographic data (EMGs) from subjects with spinal muscular atrophy (SMA). *A*: to generate a baseline for assessing the significance of our fits of the control synergies to the SMA EMGs, we fit the synergies of every control subject to the EMG of every other control, so that the resulting *R*^2^s would indicate the synergies’ generalization degree among the controls. The baseline *R*^2^s from the healthy Children group are shown here as heatmaps; each column denotes the *R*^2^s of fit for the EMG of a single subject. The *R*^2^s along the diagonal were not used in subsequent comparisons. *B*: the walking muscle synergies of the healthy children in *A* were fit to the EMGs of every subject with SMA with data collected during active-assisted motion (AAM, *n* = 14). The heat maps show the *R*^2^s from these fits using a color scale identical to that for *A*. The *R*^2^s from these control-to-SMA fits were clearly lower than the baseline *R*^2^s in *A* (*P* < 10^−4^, *t* test), thus suggesting that the muscle synergy sets underlying the SMA EMGs are different from those in the controls. *C*: comparison of the baseline *R*^2^s from fits among the control subjects (blue, left side; pink, right side; mean) with *R*^2^s from fitting the control muscle synergies to the SMA EMGs from AAM or walking. In all fits, the synergies and EMGs were age group- and side-matched. For both AAM and walking in both age groups, the control-to-control *R*^2^s were higher than the control-to-SMA *R*^2^s (**significant by multiple comparison after ANOVA with *P* < 10^−4^; *P = 0.039, *t* test).

We proceeded to assess the extent that the healthy and SMA groups share the same set of locomotor muscle synergies by fitting the synergies extracted from the walking EMGs of age-matched healthy controls to the EMGs of subjects with SMA collected from AAM or walking, with the quality of fit quantified by *R*^2^. The significance of this cross-fit *R*^2^ was evaluated against the baseline *R*^2^s expected from the interindividual synergy variability within the healthy group (69), obtained by fitting the synergies of each healthy subject to the EMGs of every other healthy subject in the same group. For the healthy Children group, the average baseline *R*^2^ across all fits (excluding the same-subject fits) was 72.6 ± 5.7% (means ± SD) for the left leg, and 71.2 ± 6.8% for the right (Fig. 4*A*); for the healthy Adult group, the average was 73.9 ± 6.2% for the left, and 73.7 ± 5.0% for the right.

Having established the baseline *R*^2^s, we next assessed whether the normative synergies of the healthy groups may generalize to describing the SMA EMGs recorded during AAM or walking. For AAM of the Children group, on both sides the *R*^2^s from fitting the synergies of the control subjects to the EMGs of every subject with SMA were clearly lower than those from fits among the controls (left, healthy-to-SMA *R*^2^ = 56.7 ± 8.1%, *P* < 10^−4^, *t* test; right, 58.9 ± 8.5%, *P* < 10^−4^, *t* test) (compare Fig. 4*A* vs. Fig. 4*B*). Similar findings were obtained for walking for both sides of both age groups (Children: left, *P* < 10^−4^, right, *P* = 0.039; Adult: *P* < 10^−4^ for left and right; *t* test; Fig. 4*C*). Thus, the normative muscle synergies for walking could not generalize to describing the SMA locomotor EMGs well.

**Figure 5.**
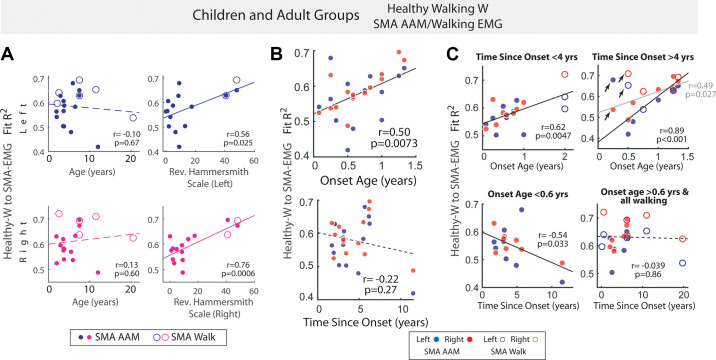
Control muscle synergies could not well-describe electromyographic data (EMGs) from early-onset spinal muscular atrophy (SMA) subjects. For every subject with (SMA), similarity of the EMGs’ structure to the normative walking muscle synergies was evaluated by fitting the walking synergies of age-matched healthy controls to the SMA EMGs from active-assisted motion (AAM) or walking, with the quality of fit quantified by *R*^2^s, and the *R*^2^s across the fits from the synergies of the control subjects averaged. *A*: the *R*^2^ of fit correlated positively and significantly with Revised Hammersmith Scale (RHS) but not with age (blue, left side; magenta, right side). *B*: the *R*^2^ of fit (AAM, both sides) correlated positively and significantly with SMA onset age (*top*) but not with time elapsed since SMA onset (*bottom*). *C*: the *R*^2^ of fit (AAM and walking, both sides) correlated positively and significantly with the SMA onset age in both subgroups of subjects with SMA recorded closer to (time since onset <4 years, *top left*) or further away from (>4 yr, *top right*) symptom onset. In the *top right*, if data from two outlier subjects (arrows) were excluded from regression, the correlation became even more robust (*r* = 0.89, *P* < 0.001; black line). Thus, the earlier the SMA onset age and the lower the residual motor function, the more the muscle synergies deviate from the normative. The *R*^2^ of fit also tended to decrease with more time elapsed since disease onset, but only in the subgroup with earlier onset age (*bottom left*). In the regressions with time since onset, all walking data were included in the subgroup with later onset age (*bottom right*) since ambulatory SMA subjects are generally those with later disease onset. All regressions in all panels were performed on data from both AAM (filled circle) and/or walking (unfilled circle) shown in the graphs. The Pearson’s *r* and its *P* value are shown on each graph. Dotted line, *P* > 0.05; solid line, *P* < 0.05 (*t* test).

We note that the *R*^2^s obtained from the healthy-to-SMA fits exhibited considerable variability across the subjects with SMA (Fig. 4*B*). This observation prompted us to ask whether any of the disease-related or demographic parameters of the subjects with SMA may account for this variability. Using regressions, we found that the healthy-to-SMA *R*^2^s of the subjects with SMA (with *R*^2^ obtained across the healthy subjects averaged for every subject with SMA) correlated positively and significantly with the RHS score (both sides) but not with the subject’s age (Fig. 5*A*). Besides, for AAM the healthy-to-SMA *R*^2^ showed a robust correlation with the age of symptom onset; the earlier the onset age, the smaller the *R*^2^ (Fig. 5*B*, *top*). This correlative trend could be observed even in subgroups of subjects with SMA recorded closer to (time since onset <4 yr) or further away from symptom onset (>4 yr), respectively (closer to onset, *r* = 0.62, *P* = 0.0047; further from onset, *r* = 0.49, *P* = 0.027; Fig. 5*C*, *top*). In the latter subgroup, if data from two subjects with SMA were excluded as outliers (Fig. 5*C*, *top right*, arrows), the correlation became even more robust (*r* = 0.89, *P* < 10^−4^). Note that the aforementioned regressions in Fig. 5*C* included *R*^2^ values from both AAM and walking, but the correlation with onset age remained significant even if only the AAM data were considered (Fig. 5*B*, *top*; *r* = 0.50, *P* = 0.0073).

We also correlated the healthy-to-SMA *R*^2^ to the time elapsed since symptom onset, in subgroups of subjects with SMA with relatively earlier (<0.6 yr) or later onset age (>0.6 yr). A significant, negative correlation was found only in the former subgroup (*r* = −0.54, *P* = 0.033) but not the latter (Fig. 5*C*, *bottom*). Thus, similarity to the normative muscle synergies declines over time only in the early-onset SMA subjects who generally display more eventual loss of motor functions than the late-onset subjects (49).

**Figure 6.**
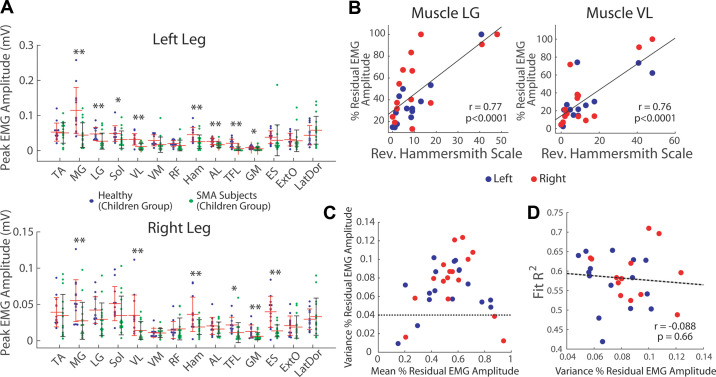
Potential roles of uneven motoneuronal losses across muscles on abnormal spinal muscular atrophy (SMA) synergies. *A*: the peak absolute electromyographic (EMG) amplitude of every muscle in every healthy (purple dot) and SMA limb (green) was estimated by finding the 99th percentile of the preprocessed, nonnormalized EMG. For many muscles of both the left (*top*) and right legs (*bottom*), the healthy-limb average (means ± SD) was significantly higher than the SMA-limb average (**P < 0.01; *P < 0.05; 1-tail *t* test), suggesting that in each SMA limb, a muscle’s peak EMG amplitude relative to the muscle’s average in healthy limbs could be a proxy for the muscle’s residual motoneuronal number and functions. *B*: correlations between the residual EMG amplitude (relative to the healthy average, in %) and the Revised Hammersmith Scale (RHS) motor functional scale across the left (blue) and right legs (red) of the SMA subjects, in muscles lateral gastrocnemius (LG) (*left*, *P* < 10^−4^) and vastus lateralis (VL) (*right*, *P* < 10^−4^), respectively. Significant, positive correlations (*P* < 0.05, *t* test for Pearson’s *r*) were observed in all muscles except adductor longus (AL), tensor fascia latae (TFL), erector spinae at L2 (ES), external oblique (ExtO), and latissimus dorsi (LatDor), suggesting that the residual EMG amplitude is a reasonable proxy for residual motoneuronal number and functions. *C*: the degree of uneven motoneuronal losses of every SMA limb was quantified by the across-muscle variance of the % residual EMG amplitude. A plot of this variance (*y*-axis) against the across-muscle mean of the residual EMG amplitude (*x*-axis) shows that limbs with very low variances had either very high or very low means, presumably reflecting motoneuronal loss at the beginning and end of the degeneration process, respectively. SMA limbs with variances <0.04 (dotted line) were excluded from subsequent analysis. *D*: the across-muscle variance of residual EMG amplitude did not correlate significantly with the *R*^2^ from the healthy-to-SMA synergy fits (*P* = 0.66). Thus, the uneven pattern of motoneuronal loss across muscles is likely not the sole factor that accounts for the abnormal SMA synergies. Sol, soleus; RF, rectus femoris; Ham, hamstrings; GM, gluteus maximus.

In addition to the onset age, another disease-related variable that may potentially account for the altered SMA synergies is the degree that the number and/or functions of the motoneurons of the different muscles are lost to different extents. The pattern and time course of motoneuronal losses across muscles are highly variable among subjects with SMA, with some presenting a loss pattern from proximal to distal muscles (49), for example. To quantify this degree of uneven motoneuronal losses, we first estimated the residual motoneuronal number and functions of each muscle of each SMA limb using the muscle’s observed peak EMG amplitude relative to that of the same muscle in the healthy controls as a rough proxy. The absolute EMG amplitude surely depends on many factors (74), but we reason that any decreases in EMG peak amplitude in subjects with SMA may reasonably be attributed to losses of motoneurons and/or their impaired discharges, rather than decreases in the single motor unit potential, given that subjects with SMA tend to have larger single motor unit potentials due to compensatory increases in motor unit sizes (75). Indeed, in our data, the SMA limbs showed reduced peak EMG amplitude in many muscles as compared with the control limbs (*P* < 0.05, 1-tailed *t* -test) (Fig. 6*A*; 8 muscles on left side; 6 on right). The reasonableness of using the residual EMG amplitude as a proxy of residual motoneuronal number and function is further supported by the positive, significant correlations between the RHS motor functional scale and the residual EMG amplitude, observed in nine of the 14 muscles (all except AL, TFL, ES, ExtO, and LatDor; *P* < 0.01 in 7 muscles, *P* < 0.05 in 2) (Fig. 6*B*).

We proceeded to quantify the degree of uneven motoneuronal losses across muscles in each SMA limb by calculating the variance, across the 14 muscles, of the residual EMG peak amplitude, and correlate this variance to the *R*^2^ from the healthy-to-SMA synergy fits. This variance is itself a dynamic variable that is expected to be low immediately after disease onset when the residual motoneuronal numbers of all muscles are uniformly high, or at the end of degeneration when the residual numbers are uniformly low. Presumably, if this unevenness impacts muscle synergy development at all, its influence should be greatest when this unevenness reaches a peak at a certain time after SMA onset. To increase the chance that the variance values we obtained, representing the state of the motoneurons at the time of EMG recording, best reflect the peak unevenness, in this correlation we considered only the limbs with variances >0.04 so that the limbs with low variances but very high or very low residual EMG amplitudes were excluded (Fig. 6*C*). The variance of residual EMG amplitude did not correlate well with the healthy-to-SMA fit *R*^2^ (*r* = −0.088, *P* = 0.66) (Fig. 6*D*). Thus, variability of synergy similarity appears to be unrelated to the variability of motoneuronal loss pattern across subjects with SMA.

### Synergy Sets from Early-Onset SMA Subjects Had Higher Dimensionality

**Figure 7.**
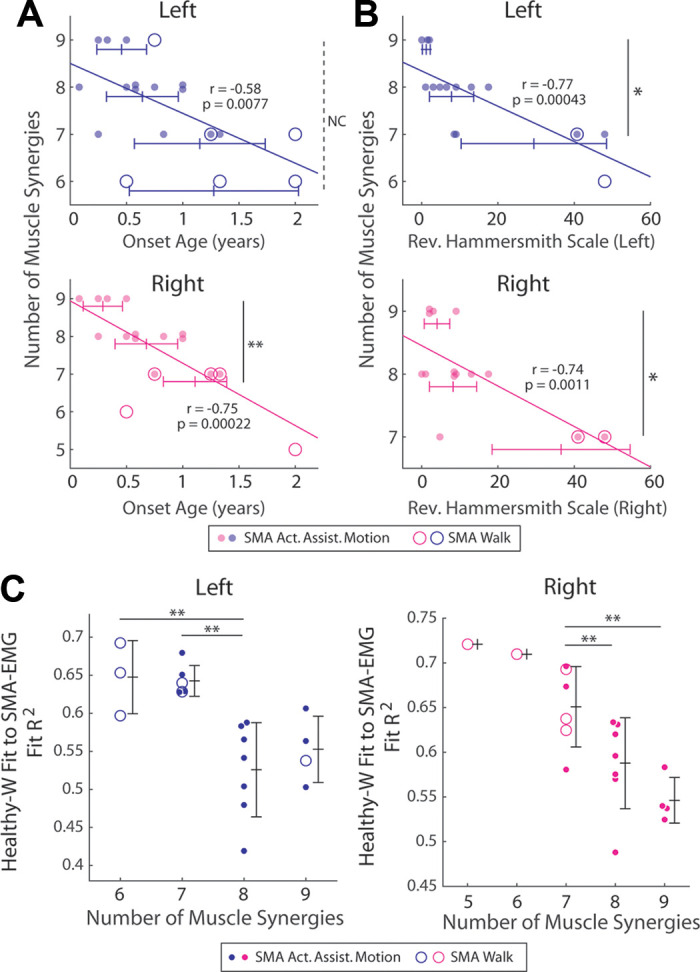
Muscle synergy sets from early-onset spinal muscular atrophy (SMA) subjects had higher dimensionality. *A* and *B*: the numbers of muscle synergies (dimensionality) of the electromyographic data (EMGs) of both behaviors of the SMA limbs were regressed against the SMA onset age (*A*) or RHS (*B*). In both regressions, a negative, significant correlation (*P* < 0.01, *t* test) was observed in both sides. Thus, the earlier the SMA onset and the lower the residual motor function, the higher the dimensionality of the EMG. All regressions were performed on data from both active-assisted motion (AAM) (filled circle) and walking (unfilled circle) presented in each graph. The Pearson’s *r* and its *P* value are shown on each graph. Across the numbers of synergies, the onset age or Revised Hammersmith Scale (RHS) (means ± SD) were also compared by multiple comparison after Kruskal–Wallis (onset age: left, *P* = 0.098; right, *P* = 0.0063; RHS: left, *P* = 0.017; right, *P* = 0.042). Significantly different pairs are indicated by the end of vertical lines (**P < 0.01; *P < 0.05; NC, *P* > 0.05). *C*: the control-to-SMA fit *R*^2^s of the SMA limbs (means ± SD) are plotted against the limbs’ dimensionality. On both sides, the higher the dimensionality, the lower the *R*^2^ of fit. These results, together with those in *A* and *B*, suggest that additional muscle synergies specific to the SMA limbs contribute to the control-SMA synergy differences in early-onset, severely impaired SMA subjects. Left, Kruskal–Wallis across all numbers of synergies, *P* = 0.0034; right, ANOVA across numbers 7 to 9, *P* = 0.0073. **significantly different pairs indicated by post hoc multiple comparison.

The aforementioned finding that the healthy synergies could not well describe the SMA EMGs—especially those recorded from early-onset subjects—implies that either the some of the SMA muscle synergies are different in structure from their counterparts in the healthy subjects, and/or the EMG of the SMA limbs are generated by a higher number of muscle synergies than that underlying the healthy EMGs. We first investigated the latter possibility by extracting muscle synergies from the EMGs of every SMA limb and examined the numbers of synergies (or dimensionalities) across limbs. Combining the dimensionalities from both AAM and walking, we found that the average dimensionality of the SMA limbs (left, 7.53 ± 0.96; right, 7.67 ± 1.08) was higher than that of the age-matched healthy limbs (left, 6.31 ± 0.63; right, 6.85 ± 0.80) (left, *P* = 9.6 × 10^−4^; right, *P* = 0.022; Mann–Whitney). Across subjects with SMA and both behaviors, the higher the EMG dimensionality, the lower the *R*^2^ from fits with the healthy synergies (Fig. 7*C*), and the lower the onset age (Fig. 7*A*) and RHS score (Fig. 7*B*), consistent with the *R*^2^ results aforementioned (Fig. 5). Hence, additional muscle synergies unique to the SMA limbs contribute to the differences between the normative and post-SMA synergy sets; the early-onset subjects with lower residual motor functions also tended to have more additional SMA-specific synergies expressed in their limbs.

### The Additional SMA-Specific Synergies Had Active Muscles in Multiple Body Segments

We proceeded to further examine the characteristics of the additional synergies unique to the SMA limbs. To obtain the additional synergies, for each SMA limb, we fit the synergies of age- and side-matched controls to the SMA EMG using our modified NMF algorithm. Our algorithm implements synergy fitting while allowing concurrent extraction of a predefined number of additional synergies (*N^add^*) from the EMGs as well as concurrent updating of a predefined number of the original synergies being fit (*N^up^*). During fitting, *N^up^* and then *N^add^* were increased successively until these numbers resulted in the *R*^2^s of fits not smaller than the baseline *R*^2^s at identical *N^up^* (N^up^* and N^add^*) (see materials and methods; Tables 2 and 3). The additional SMA-specific synergies were then represented by the N^add^* synergies extracted. For all SMA limbs with N^add^* > 0, the additional synergies derived from fits with the synergies of all healthy subjects were then collected and *k*-means-clustered separately for each side. The cluster centroids from both sides were then matched by scalar-product similarity.

**Table 2.** The optimal N^up^ and N^add^ when control walking muscle synergies were fit to AAM EMGs of subjects with SMA

Age Group	Subject Number	Left		Right	
		N^add^*	N^up^*	N^add^*	N^up^*
Children	1701	2	0	1	0
	1702	2	0	2	0
	1703	1	4	1	3
	1704	1	0	2	0
	1705	2	0	1	1
	1706	2	0	2	0
	1710	1	1	0	4
	1711	1	0	0	0
	1712	1	0	0	1
	1713	1	0	1	0
	1714	2	1	1	0
	1715	2	0	1	0
	1716	2	0	0	4
	1717	2	0	1	0

AAM, active assisted motion; EMGs, electromyographic data; N^add*^, optimal number of additional synergies; N^up*^, optimal number of updated synergies; SMA, spinal muscular atrophy.

**Table 3.** The optimal N^up^ and N^add^ when control walking muscle synergies were fit to walking EMGs of subjects with SMA

Age Group	Subject Number	Left		Right	
		N^add^*	N^up^*	N^add^*	N^up^*
Children	1501(1)	0	2	NA	NA
	1501(2)	1	0	0	0
	1605	0	1	0	0
	1711	1	0	0	1
	1712	0	1	0	0
Adult	1602	1	0	0	1

NA, electromyographic (EMG) data not available; N^add*^, optimal number of additional synergies; N^up*^, optimal number of updated synergies; SMA, spinal muscular atrophy.

**Figure 8.**
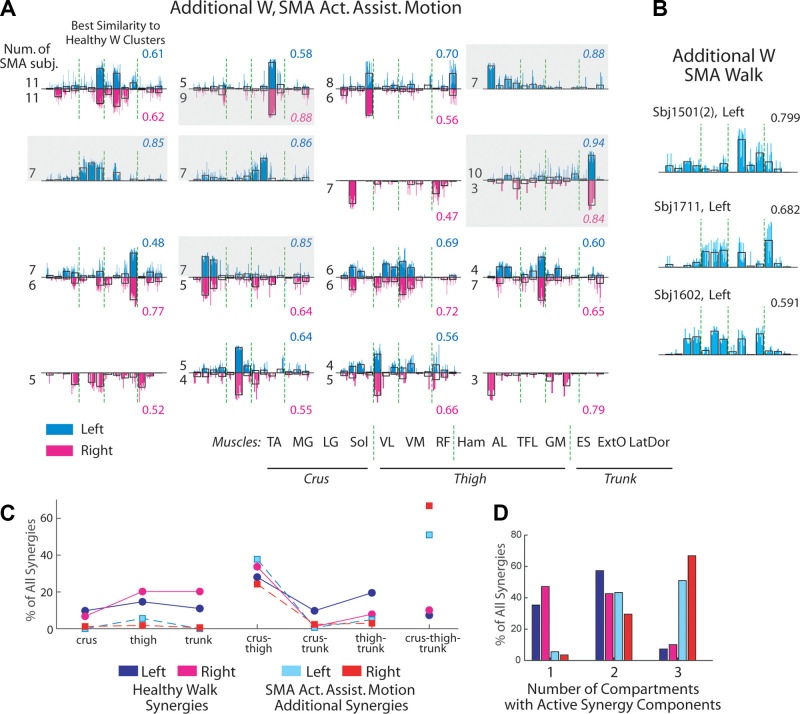
The additional spinal muscular atrophy (SMA)-specific muscle synergies had active muscle components in multiple limb segments. *A*: for every SMA limb, the number of additional synergies needed to describe the active-assisted motion (AAM) electromyography (EMG) at baseline level (N^add^*) was determined (Table 2). All SMA-specific additional synergies for limbs with N^add^* > 0 from all control-to-SMA fits were collected and *k*-means clustered (left-limb synergies, blue bars, *n* = 286; right-limb, magenta bars, *n* = 169). The clusters of the two sides were paired up by best-matching scalar product (SP) between cluster centroids (>0.8) (uncolored bars) and each matched pair are shown in the same graph. The centroid of every cluster was also compared with the side-matched control synergy cluster centroids (Fig. 3) (SP shown on the graph’s right) and those with SP > 0.8 (gray shaded clusters) are excluded from further analysis. The number of SMA subjects carrying an AAM synergy from each cluster is shown on the graph’s left. Note that many non-gray-shaded clusters have activation components spanning muscles in multiple limb or body segments (crus, thigh, and trunk). *B*: the SMA-specific additional synergies for walking for the three limbs with N^add^* > 0 (Table 3) from all control-to-SMA fits (*n* = 34), shown individually for each limb. The number at top right corner indicates best-matching SP between the synergies’ mean (uncolored bars) and age- and side-matched control synergy centroids. *C*: the percentages of muscle synergies with activation components in muscles of any one, any two, or all three of the limb/body segments (crus, thigh, trunk), respectively, for the additional SMA-specific synergies for AAM (left, cyan; right, red; dotted line) and healthy control synergies for walking (Children group; left, blue; right, magenta; solid line). Components were defined as active if the component magnitude, after *l*^2^ normalization of the muscle synergy vector, is >0.1. The percentages of SMA-specific synergies with components in three segments are clearly higher than those of control synergies. *D*: same data as *C*, except that the total percentage for each number of limb/body segments (1 to 3) with active synergy components is presented.

For AAM, we identified 26 clusters of additional SMA-specific synergies from both sides with 13 clusters from each side (Fig. 8*A*). To further ensure that the additional synergies analyzed could only be observed in SMA but not the healthy subjects, we earmarked those clusters with centroids similar to those of the synergies of age- and side-matched healthy controls (Fig. 8*A*, gray shaded synergies), thus resulting in 19 clusters that were genuinely SMA specific. When compared with the control synergies, the SMA-specific synergies appeared overall to be less sparse, with components in muscles of multiple limb or body segments (e.g., clusters in row 4, column 3 of Fig. 8*A* have components in crus, thigh, and trunk muscles). The same is to be said for the additional SMA-specific synergies identified from walking (Fig. 8*B*).

We calculated the percentages of the SMA-specific synergies with activations in muscles of only the crus, thigh, or trunk, in any two of the aforementioned segments, and in all three segments, and compared them with the percentages in the healthy control synergies (Fig. 8*C*). Clearly, on both sides, the majority of SMA-specific synergies had components in muscles of two or three segments while most of the control synergies, in one or two segments. In fact, more than half of the SMA-specific synergies contained activations in muscles of all three body segments (Fig. 8*D*).

### Synergies Modified in Subjects with SMA Had Active Muscles in Multiple Body Segments

As mentioned earlier, besides the additional synergies unique to the SMA limbs, other healthy-SMA synergy differences may originate from some synergies that are expressed in the healthy limbs but structurally modified in the SMA limbs. In our NMF implementation, these altered synergies are captured by the N^up^* synergies that are modified from a selected subset of the original healthy synergies fit to the SMA EMG. For all SMA limbs with N^up^* > 0 (Tables 2 and 3), we collected all updated synergies derived from all fits of both sides and grouped them into clusters (with *k*-means implemented on the original healthy synergies associated with the updated synergies). The pre- and postupdate synergies were then compared for every cluster.

**Figure 9.**
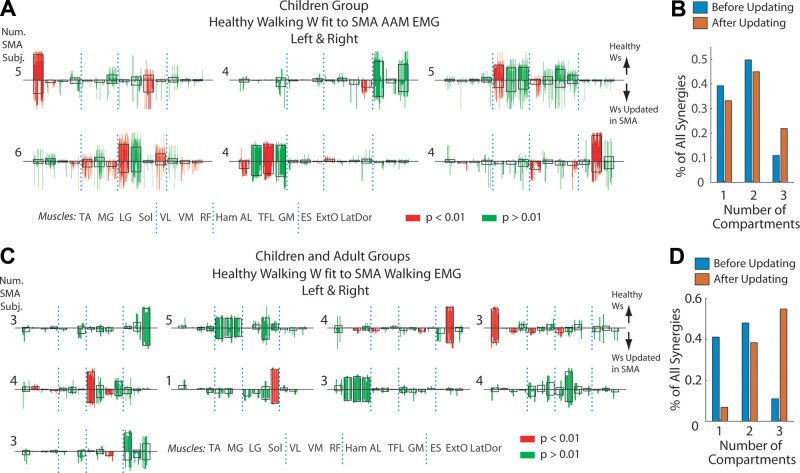
Synergies modified in subjects with spinal muscular atrophy (SMA) had active muscle components in multiple limb segments. *A*: for every SMA limb, in addition to N^add^* (Fig. 8), the number of original control synergies updated during the fit to describe the active-assisted motion (AAM) electromyographic data (EMGs) at baseline level (N^up^*) was determined (Table 2). All pre- and postupdate synergies from all control-to-SMA fits of all limbs with N^up^* > 0 (*n* = 247, left and right sides combined) were collected, and the preupdate synergies were categorized by *k*-means into six clusters. In each cluster, muscle components of the *l*^2^-normalized preupdate synergies (from healthy subjects; upward bars) and postupdate synergies (updated for SMA EMG; downward bars) were then compared muscle by muscle with the *t* test or Mann–Whitney test (red, *P* < 0.01; green, *P* > 0.01). Like the SMA-specific additional synergies, the postupdate synergies tended to have activations in muscles spanning more limb/body segments. The number of SMA subjects carrying a synergy in each cluster is shown on the graph’s left. *B*: the percentages of muscle synergies with active muscles in 1 to 3 limb/body segments (crus, thigh, trunk), before (blue) and after (red) updating, for the muscle synergies in *A*. Definition of active muscle components is the same as that stated for Fig. 8*C*. Note that the percentage of three-segment synergies after updating doubles that before updating. *C*: similar to *A*, but for the SMA limbs with walking EMGs collected (Table 3). The synergies from all SMA limbs (left or right sides) in both age groups with N^up^* > 0 (*n* = 73) were combined for this analysis. As in *A*, the postupdate synergies tended to have activations in muscles spanning more limb/body segments. *D*: the percentages of muscle synergies with active muscles in 1 to 3 limb/body segments before (blue) and after (red) updating, for the muscle synergies in *C*. Note the fivefold increase in the percentage of three-segment synergies after updating.

For AAM, we identified six clusters of muscle synergies modified in SMA (Fig. 9*A*). None of the pre-to-post-update differences in these clusters involved the individuation of muscles AL and TFL, the primary difference between the walking and AAM synergies observed earlier in the two healthy subjects with EMGs collected from both motor tasks (Supplemental Fig. S1, *D* and *E*). Thus, the synergy alterations in the postupdate synergies here should likely be due to SMA pathophysiology rather than how the synergies of the two behaviors naturally differ in all subjects. Notably, in these clusters the postupdate synergies for the SMA EMGs appeared to involve muscles in more body segments than their preupdate counterparts (e.g., *cluster 4* in Fig. 9*A*, row 2, column 1). After updating, the percentage of synergies with muscles of all three segments doubled (Fig. 9*B*).

For walking, we identified nine clusters from the modified synergies of both sides of both age groups (Fig. 9*C*). Similar to AAM, the postupdate synergies appeared less sparse than their associated preupdate synergies. After updating, more than half of the synergies contained components in muscles of all three segments (Fig. 9*D*).

## DISCUSSION

In this study, we characterized the locomotor muscle synergies of a cohort of patients with SMA types II or IIIa with diverse clinical presentations (Table 1) and compared them against the synergies of age-matched healthy controls (Fig. 3), with the goal of gaining insights into the potential roles of early motor experience, corticospinal activities, and motor compensation in the expression of locomotor synergies. We found that the healthy synergies could not adequately account for the EMGs of the subjects with SMA (Fig. 4) and that the earlier the SMA onset age and the lower the subject’s residual motor function, the more different the synergies of the SMA subject were from the normative (Fig. 5). By manipulating the NMF, we identified the healthy synergies that were altered in subjects with SMA (Fig. 9, *A* and *C*) and the additional SMA-specific synergies (Fig. 8, *A* and *B*), both of which contributed to the healthy-SMA differences in EMG structure. The SMA-specific synergies also comprised active components in muscles of more body or limb compartments than the normative synergies (Fig. 8*D* and Fig. 9, *B* and *D*). Overall, our results provide clues into the origin of abnormal neuromotor coordination in subjects with SMA in relation to the normative developmental process of locomotor muscle synergies in infants and toddlers.

### Muscle Synergies Reflect the Motor Functional Status of Subjects with SMA

The most salient finding of this study is that subjects with SMA expressed lower limb muscle synergy sets that were overall different from those observed in the controls. In our synergy comparison, we first fit the healthy synergies to the EMGs of the subjects with SMA and found that the quality of this fit, quantified by *R*^2^, was below that expected from the baseline intersubject synergy variability (Fig. 4), but this drop in the *R*^2^ of fit could be rescued with the concurrent extraction of SMA-specific synergies during fitting (Table 2). Thus, the EMGs of the SMA group contain both structures that can be found in the control group, and structures not found in the control group that are represented by the additional and modified synergies extracted (Fig. 4*C*). The original *R*^2^ of fit for each SMA subject (Fig. 4*B*) naturally indicates the distance between the subject’s synergy set and the normative muscle synergies; the lower the *R*^2^, the more different the subject’s synergies are from the normal synergies.

For any subjects with SMA, high similarity of the subject’s synergies to the normative synergies should reflect a higher level of overall motor functions. This is directly supported by our finding that across subjects with SMA, the similarity of the SMA synergy sets to those of the healthy controls, as indicated by the aforementioned *R*^2^ of fit, correlated positively with the subjects’ RHS score (Fig. 5*A*). The same similarity value correlated positively with the disease onset age in both subject subgroups recorded either closer to or further away from symptom onset (Fig. 5*C*, *top*), consistent with the well-known generality that the earlier the age of SMA onset, the more severe the symptoms (49, 78). Also, similarity to the normative synergies decreased as the time elapsed since disease onset increased, but only in the subject subgroup with earlier SMA onset and not in those with later onset (Fig. 5*C*, *bottom*); this again agrees well with the previous finding that motor functions of subjects with SMA deteriorate over time, with the rate of loss being more pronounced in patients with earlier onset age (49, 79). The good alignment between our similarity results of the SMA synergies and the natural history of SMA suggests that the lower-limb muscle synergies of subjects with SMA may potentially serve as a marker that indicates their neurological and motor functional states, analogous to how synergies may assess motor functions for stroke (80, 81), cerebral palsy (82, 83), and other disorders (84).

### The Normative Synergies Preserved in Subjects with SMA

In our cross-fitting analyses for both the AAM and walking tasks, the muscle synergies of the age-matched controls could account for ∼50% of the EMG variance of the subjects with SMA, on average (Fig. 4*C*). This suggests that at least a subset of the normal locomotor synergies is preserved in the subjects with SMA. Given that the number of synergies of the control limbs was ∼6–7 and that N^up^* of the SMA limbs could be up to 3–4 (Table 2), we estimate that 3–4 of the synergies may be shared by the control and SMA limbs. These preserved synergies are generally consistent with the idea that there exist evolutionarily conserved locomotor modules (16, 21) that are determined very early in life (23). Precursors of these synergies may be identified in neonatal behaviors such as stepping (20), with these synergies’ eventual representations being fractionations of their precursors (20, 27, 32). Sensory and descending activities during development may serve to facilitate such fractionations (40) and to fine-tune their structures (23, 41). For our subjects with SMA, the developmental sensory and corticospinal activities and other intraspinal mechanisms before or around symptom onset may already be sufficient to ensure the emergence of the preserved synergies, as suggested by the finding in a rodent SMA model that locomotor-like rhythms could be elicited by spinal stimulations at the earliest SMA stage (but not necessarily at later stages) (59). The preserved synergies are then robustly expressed despite the abnormal sensory and descending activities after SMA (see *SMA-Specific Synergies as Possible Developmentally Abnormal Synergies*). Recent data from human patients with spinal cord injury have revealed that the spinal cord likely encodes a set of locomotor synergies as a “common core infrastructure” that is subject to modification from sensory, descending, and voluntary drives (41). The control synergies preserved in our SMA subjects may well be parts of this spinal “core” that are under the influence of the residual sensory and descending systems.

As to the SMA-specific synergies, we will devote the rest of the Discussion to their multiple possible origins, which are more enigmatic.

### SMA-Specific Synergies as Possible Developmentally Abnormal Synergies

Another notable finding of this study is that the SMA-specific synergies, be they identified as modified or additional synergies, were composed of muscles that spanned more body or limb compartments than the normative synergies (Figs. 8 and 9). These multicompartment muscle synergies are reminiscent of the locomotor synergies observed in human infants and toddlers, which often feature cocontractions of antagonistic muscles crossing multiple joints (26, 43). For instance, one of the two neonatal stepping synergies in Dominici et al. (their Fig. 2C) (21) and Sylos-Labini et al. (their Supplemental Fig. S5) (20) comprises 10 muscles in the thigh and crus. Stepping synergies with coactivations of biceps femoris (BF) and RF in the thigh as well as LG in the crus have also been identified in neonates, infants, and toddlers (20, 27, 44). As toddlers further develop into preschoolers (age 2–4), the ankle extensors in the crus gradually separate from the thigh muscles to become an independent synergy (21). More generally, it has been proposed that the nonsparse synergies that are elicited during neonatal stepping are locomotor precursors (20), inborn or determined very early in life (23), whose gradual fractionation would give rise to the relatively sparse locomotor synergies of adults (20, 27, 32). These prior works have hinted at the possibility that synergy fractionation reflects and results from the early sensorimotor exploration and experience necessary for molding the synergies into modules suitable for controlling the growing limb with changing biomechanical properties (32, 44, 85), and for diversifying the bipedal tasks accomplishable.

We speculate that the multicompartment SMA-specific synergies observed in our SMA preschoolers and children are modules that are developmentally abnormal or immature for the subjects’ ages and originate from the nonsparse synergies from an earlier age. These SMA-specific synergies may be produced, for example, by incomplete or delayed fractionations of the precursor synergies, or other fractionation patterns that differ substantially from those in typically developing children. Assuming that there exists for humans a critical period during which fractionation of the precursor synergies is particularly sensitive to sensorimotor activities (86, 87), faulty synergy fractionation can occur when, during this period, the patterns and amount of daily movement are deranged, and the sensory, corticospinal, and intraspinal activities are impaired due to motoneuronal degeneration, loss of sensorimotor synapses, and dysfunction of other networks from SMA (52, 53). Our finding that the subjects’ synergy similarity to the normative decreased with earlier onset age (Fig. 5, *B* and *C*) supports this interpretation. The earlier the disease onset, the more overlap of the postonset time with the critical period, and for a longer duration synergy fractionation would proceed under sensorimotor disruption. In typically developing children, since much of the normal fractionation would have occurred by the preschooler stage (age 2–4) (21), this presumed critical period may happen between birth (46) and age 2, a period that coincides perfectly with the range of onset age in our cohort (Table 1).

**Figure 10.**
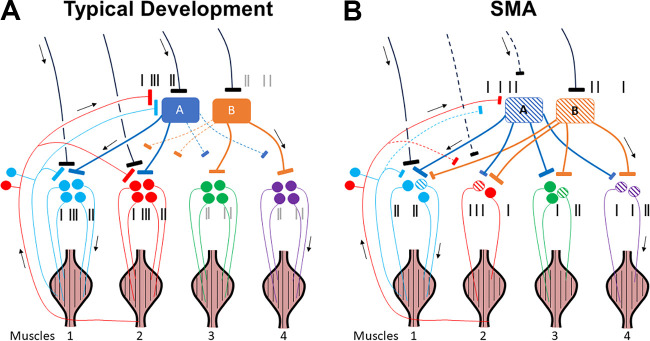
One hypothetical mechanism of muscle synergy fractionation in typically developing children and children with spinal muscular atrophy (SMA). *A****:*** during normal development, the spinal interneurons (*A* in blue, *B* in orange) that encode the precursor muscle synergy (coactivating muscles 1 to 4) compete for synaptic spaces on the motoneuronal pools of the same muscles (neurons in cyan, red, green, and purple, for muscles 1 to 4, respectively) in an activity-dependent manner. During early exploratory or voluntary movement, any interneuron (e.g., *A*) and the motoneurons of a subset of muscles within the precursor (e.g., muscles 1 and 2) are synchronized (black spikes) through reinforcements from proprioceptive (cyan and red lines from muscles 1 and 2, respectively) and descending inputs (solid black lines) to the motoneurons and interneurons as the same subset of muscles are coactivated. Such synchronizations strengthen the interneuron’s connections to the reinforced motoneurons, allowing these axons to outcompete the innervations to the same muscles from another interneuron (dotted orange line denoting retracting axons from *B*). The interneuron’s connections to the other unreinforced muscles in turn are outcompeted by other synchronizations (gray spikes). This way, the original precursor synergy is fractionated into two new synergies (muscles 1 and 2 from *A*, 3 and 4 from *B*). Arrows indicate the direction of information flow. *B*: after SMA onset, the combined effects of motoneuronal degeneration, motoneuronal, and interneuronal dysfunction (neurons with diagonal stripes), and losses of proprioceptive and descending inputs (dotted line) lead to reduced and deranged exploratory and voluntary movement while disrupting any activity synchronizations seen in normal children. The lack of activity-dependent competitions between interneurons results in failed or incomplete fractionation of the precursor muscle synergy.

How exactly may SMA-related sensorimotor abnormalities lead to failed or faulty synergy fractionation, at least hypothetically? First, the precursor muscle synergies that activate many leg muscles are likely encoded by spinal premotor interneurons that innervate the motoneuronal pools of the same muscles in the synergies (8, 88, 89). In a typically developing child, exploratory and/or voluntary movement patterns that are repeatedly performed during critical period would lead to activity synchronization between a synergy-encoding interneuron and the motoneurons of a certain subset of muscles within the precursor synergy that are activated together. This synchronization may be reinforced by sensory activities arising consistently from that subset of muscles through proprioceptive-motor connections, as well as corticospinal and intraspinal activities that originally produce the movement. Connections between the interneuron and the motoneurons of this muscle subgroup are then strengthened through activity-dependent plasticity whereas the interneuron’s connections to the other muscles without concurrent reinforcement are eliminated as they are outcompeted by other synchronizations involving other interneurons (Fig. 10*A*). With these synaptic reinforcements and pruning, the synchronized subgroup of muscles, as a new muscle synergy, are then gradually dissociated (i.e., fractionated) from the other muscles within the precursor synergy.

After the onset of SMA, not only are the limb muscles gradually denervated with motoneuronal degeneration happening faster for some muscles than the others, but on the surviving motoneurons there is also a reduction in the number of proprioceptive synapses (52, 90) with the loss of afferent synapses preceding the motoneurons’ eventual degeneration (53). Concurrently, there may be a decrease in corticospinal inputs to the spinal cord because of the degeneration (55) or functional losses of motor cortical and/or cerebellar neurons (57). The functional properties of the spinal interneurons, their interactions with the descending drives, and the capacity of the descending systems to compensate for any motor deficits may also be compromised (60). With the combined effects of reduced and abnormal movement owing to motoneuronal losses, the partial deafferentation of the remaining motoneurons, and the abnormal descending and intraspinal activities arising from SMA pathophysiology, any movement during the critical period may produce insufficient activity synchronization between the interneurons and motoneurons to result in adequate activity-dependent plasticity for the proper sculpting of the interneuronal circuit (Fig. 10*B*). This then leads to failed or incomplete fractionation of the precursor muscle synergies, a scenario analogous to the classic finding that geniculocortical afferents from the two eyes would fail to segregate into ocular dominance columns if retinal inputs are blocked binocularly (91). The aforementioned hypothetical mechanism surely demands additional validations, but it highlights the perspective that motor functional impairment after SMA is not simply the outcome of motoneuronal degeneration, but rather that of a dysfunctional sensorimotor circuit that emerges from the consequences of motoneuronal, afferent, and other losses during a critical time of motor development (52, 92).

### Synergy Alterations in SMA Independent of Sensorimotor Inputs to Spinal Cord

Our interpretation of the SMA-specific synergies outlined earlier stresses the combined contributions of abnormal sensorimotor experience, altered descending activities, and other SMA-induced changes of the spinal circuits to the emergence of the abnormal synergies. Since we do not know the relative importance of these factors in the abnormal development of the SMA synergies, it is well possible that all synergy changes observed here arise solely from the SMA-induced alterations in the neuronal properties and developmental dynamics of the interneuronal and motoneuronal circuits, in a way that is independent of the abnormal sensorimotor inputs to the spinal cord. Specifically, faulty fractionation of the precursor synergies may be driven only by the uneven pattern of motoneuronal loss across muscles (49, 73, 93) so that, for instance, for a premotor interneuron, its connections to only the muscles with motoneuronal pools presenting adequate trophic support to the innervating synapses are retained while the others are pruned. Although we cannot exclude this possibility, we observed no significant relationship between the SMA-to-healthy synergy similarity and the across-muscle variance of the residual EMG amplitude (Fig. 6*D*), thus suggesting that the unevenness of motoneuronal losses is likely not the sole factor that drives abnormal synergy development in SMA. It must be acknowledged that our method of estimating the relative degree of motoneuronal degeneration for each muscle is rather crude, even though the residual EMG amplitude of 9 of the 14 muscles did correlate significantly with the RHS motor functional scale (Fig. 6*B*). A full examination of how the diverse patterns of motoneuronal vulnerability to degeneration impacts synergy development would require longitudinal recordings and more sophisticated techniques of estimating the motoneuronal losses of individual muscles.

Beyond motoneuronal losses, the observed SMA-specific synergies may also arise from the uneven alterations of the recruitment patterns of the remaining motoneurons across muscles. After SMA, the surviving motoneurons become hyperexcitable (60, 94). The number of excitatory synapses onto the motoneurons from both afferent (52, 53, 95) and central sources (96) are also consistently reduced to the extent that the motoneurons’ hyperexcitability may not adequately compensate for (53), thus making them more difficult to be recruited (59). It is conceivable that after SMA, the relationships between motoneuronal inputs and their recruitment levels are changed unevenly across the motoneuronal pools of the muscles, so that the across-muscle EMG correlations imposed by the original synergies are weakened, leading NMF to identify altered synergies that are likely more fragmented than the original ones. Since the SMA-specific synergies we observed here have active components from muscles of more limb compartments (Fig. 8*D* and Fig. 9*D*), we believe such alteration of the motoneuronal recruitment curves is unlikely to be the sole factor that accounts for the SMA-specific synergies. But how complex changes of motoneuronal recruitment patterns may impact the identifiability of muscle synergies from EMGs and the performances of different extraction algorithms deserves to be more comprehensively evaluated in simulated and experimental data sets in future studies [perhaps à la Tresch et al. (7)].

Other possibilities that may account for the abnormal SMA muscle synergies include any functional or connectivity impairment of the spinal interneurons caused either directly by SMN depletion within the interneurons, or as a consequence of motoneuronal degeneration and deafferentation. In a *Drosophila* model of SMA, the mutant phenotype can only be reversed after SMN is restored in both proprioceptive and central cholinergic neurons (90); thus, some defects of spinal cholinergic interneurons induced by SMN insufficiency may contribute to the neural circuit dysfunction that underscores the post-SMA motor impairment. Indeed, in the mouse spinal cholinergic interneurons that modulate locomotor motoneuronal activity have been identified (97). Also, in mice with severe SMA, ventral horn interneurons exhibit greater excitability and increased frequency of spontaneous inhibitory postsynaptic potential (60). It must be emphasized that many of these interneuronal findings were derived from animals genetically modified to model the severe SMA types whose neuropathological presentations are different from those of the milder types (49). To what extent these abnormalities are present in humans with milder SMA forms, such as the patients studied here, and whether they contribute to functional impairment or are just adjustments that compensate for other dysfunctions are questions that await future research.

### Potential Roles of Sensory and Descending Activities in Typical and Post-SMA Synergy Development

Assuming that the SMA-specific synergies observed here are not just the results of input-independent changes within the spinal cord, our results then imply that sensory afferent and descending inputs to the spinal cord probably contribute to the typical developmental expression of locomotor synergies. Such patterned inputs may help sculpt the neonatal synergies, through fractionation, for the subsequent execution of locomotion. This view aligns well with the recent demonstrations of synergy fractionation and the accompanying increase in the number of modules during child-to-adult locomotor development (20, 21, 27, 32, 44). It has been suggested that the spatial synergies for neonatal stepping and the temporal patterns observed in neonatal kicking are two distinct locomotor precursors (20). It is possible that both the fractionation of the spatial synergy precursors and the linkage of the fractionated spatial synergies with their corresponding temporal activations are underpinned by reconfiguration of the spinal circuits as directed by sensorimotor inputs to the spinal cord. The details of this circuit reconfiguration remain elusive.

Concerning the roles of the sensory afferents, we do not know the exact biomechanical events and behaviors that should best facilitate synergy fractionation with their afferent patterns. As has been previously suggested, the feedback elicited when the feet contact a support surface during neonatal stepping (44) and postural feedback associated with weight bearing (25) are likely important for synergy expression. Indeed, for our subjects with SMA, their lack of any substantial sitting or standing experience throughout their development may specifically contribute to their abnormally fractionated synergies (Figs. 8 and 9). Alternatively, since the feedback generated by spontaneous muscle twitches during sleep or fetal movements can lead to the emergence of spinal circuits that reflect the limb biomechanical properties (19, 98, 99), any spontaneous infantile movements before walking onset may be critical as well.

Concerning the roles of the descending system, animal studies have shown that both the distributions of the different spinal interneuronal types (37, 38) and the detailed configurations of the spinal sensorimotor circuits (36) are dependent on developmental corticospinal activities. During this process, both the cortical and spinal circuits are reorganized so that supraspinal and afferent projections can be functionally integrated into the spinal circuits. In human toddlers, activations of the two locomotor synergies that emerge around the onset of independent walking cohere with motor cortical activities (40). If descending activities participate in the developmental sculpting of these two synergies, as the child grows up into adulthood, the same descending axons may continue to recruit or activate these synergies (100) and/or fine-tune or augment their spatial structures (23). As such, the SMA-specific synergies reported here may also originate from the failure of the descending inputs to cooperate with the spinal circuits to produce the intact fine-tuned synergies, due either to degeneration of cortical neurons (55) or decreases in the cortical neurons’ excitability following SMA (57), and/or to other changes in the properties of the spinal interneurons (60). Alternatively, as argued earlier, the abnormally fractionated synergies could potentially result from reduced corticospinal participation in the developmental configuration of the spinal circuits (Fig. 10*B*). But many of these cortical abnormalities were only documented in animals with severe SMA or human patients at the latest stages of the disease. Their presence and relevance to neural development at the earlier stages of patients with milder SMA await further confirmations.

Of note, after the critical period, the motor cortex may continue to adapt itself to utilize the normal and abnormal synergies encoded in the spinal cord. In patients with SMA types III/IV adult, neuroimaging has revealed increased gray matter density in the motor cortex (101) suggestive of reorganization of cortical circuits. Such reorganizations may also reflect the activation and/or acquisition of corticospinal and corticoreticulospinal mechanisms that drive motor strategies for compensating for any motor deficits resulting from motoneuronal losses or abnormal synergies, perhaps reminiscent of compensation-driven plasticity observed in stroke survivors (102). A subset of the SMA-specific synergies found here may well be motor modules for such motor compensations. They may or may not be acquired through motor learning.

### Conclusion

In summary, the locomotor muscle synergies of subjects with SMA have complex origins. The synergies shared by the SMA and control subjects may reflect components of a core spinal modular infrastructure that is modified by sensorimotor inputs, and shaped by genetic processes and/or early sensory and descending activities. The SMA-specific synergies may arise from multiple processes, including activity-dependent developmental plasticity from altered sensory and descending drives (Fig. 10*B*), SMA-induced changes of spinal interneuronal properties and intraspinal developmental dynamics, altered interactions between the cortical and spinal networks, and compensatory mechanisms driven by descending or voluntary drives. Also, methodological limitations from the potential uneven across-muscle changes in motoneuronal recruitment pattern after SMA cannot be excluded.

More generally speaking, our interpretations of the shared and SMA-specific synergies are consistent with the view that muscle synergies observed later in life possess structures that incorporate the sensorimotor history experienced earlier in life. According to this view, muscle synergies are motor solutions sculpted by the interactions between genetically determined processes and the sensorimotor inputs to the spinal cord generated during early spontaneous and voluntary movements. These solutions are then encoded within a “core spinal infrastructure” (2, 8–11, 23, 41, 88), used subsequently for the construction of motor behaviors (3–6), and are subject to being further adjusted (23, 41). This view is consistent with the proposal that muscle synergies undergo long-term plasticity during development (32), acquisition of novel motor skills (32, 33, 103), aging (104), and recovery from injuries (80, 105).

## DATA AVAILABILITY

Data will be made available upon reasonable request.

## SUPPLEMENTAL DATA

10.6084/m9.figshare.24978804 Supplemental Text and Supplemental Fig. S1: http://doi.org/10.6084/m9.figshare.24978804.

## GRANTS

This work was supported by the National Science Foundation of China-Hong Kong Research Grants Council Joint Research Scheme under Grant N_CUHK456/21, the Hong Kong Research Grants Council Grants 24115318, R4022-18F, 14114721, and 14119022, and the CUHK Grants FIA2016/A/04, rsfs1819/0604/19hc, and grs1819/0426/19hc (to V.C.K.C.).

## DISCLOSURES

No conflicts of interest, financial or otherwise, are declared by the authors.

## AUTHOR CONTRIBUTIONS

V.C.K.C., S.C.W.H., Y.T., L.W., D.L., and R.T.H.C. conceived and designed research; V.C.K.C., S.C.W.H., J.H.Z.-L., Z.Y.S.C., Y.T., and R.T.H.C. performed experiments; V.C.K.C., S.C.W.H., Y.T., and G.Y. analyzed data; V.C.K.C., S.C.W.H., Y.T., and R.T.H.C. interpreted results of experiments; V.C.K.C. prepared figures; V.C.K.C., S.C.W.H., and Y.T. drafted manuscript; V.C.K.C., J.H.Z.-L., and R.T.H.C. edited and revised manuscript; V.C.K.C., S.C.W.H., J.H.Z.L., Z.Y.S.C., Y.T., G.Y., L.W., D.L., and R.T.H.C. approved final version of manuscript.
